# Epigenetic associations with kidney disease in individuals of African ancestry with *APOL1* high-risk genotypes and HIV

**DOI:** 10.1093/ndt/gfae237

**Published:** 2024-10-24

**Authors:** Rachel K Y Hung, Ricardo Costeira, Junyu Chen, Pascal Schlosser, Franziska Grundner-Culemann, John W Booth, Claire C Sharpe, Kate Bramham, Yan V Sun, Vincent C Marconi, Alexander Teumer, Cheryl A Winkler, Frank A Post, Jordana T Bell

**Affiliations:** Department of HIV and Sexual Health, King's College Hospital, London, UK; Department of Twin Research & Genetic Epidemiology, King's College London, London, UK; Department of Twin Research & Genetic Epidemiology, King's College London, London, UK; Department of Epidemiology, Rollins School of Public Health, Emory University, Atlanta, GA, USA; Institute of Genetic Epidemiology, Faculty of Medicine and Medical Center, University of Freiburg, Freiburg, Germany; Department of Epidemiology, Johns Hopkins Bloomberg School of Public Health, Baltimore, MD, USA; Institute of Genetic Epidemiology, Faculty of Medicine and Medical Center, University of Freiburg, Freiburg, Germany; Department of Renal Medicine, Bart's Health NHS Foundation Trust, London, UK; Department of Renal Medicine, King's College Hospital NHS Foundation Trust, London SE5 9RS, UK; Department of Renal Medicine, King's College Hospital NHS Foundation Trust, London SE5 9RS, UK; Department of Epidemiology, Rollins School of Public Health, Emory University, Atlanta, GA, USA; Atlanta Veterans Affairs Health Care System, Decatur, GA, USA; Atlanta Veterans Affairs Health Care System, Decatur, GA, USA; Department of Global Health, Rollins School of Public Health, Emory University, Atlanta, GA, USA; School of Medicine, Emory University, Atlanta, GA, USA; DZHK (German Centre for Cardiovascular Research), Partner Site Greifswald, Greifswald, Germany; Department of Psychiatry and Psychotherapy, University Medicine Greifswald, Greifswald, Germany; Department of Population Medicine and Lifestyle Diseases Prevention, Medical University of Bialystok, Bialystok, Poland; Basic Reseach Program, Frederick National Laboratory for Cancer Research and the Cancer Innovation Laboratory, Center for Cancer Research, National Cancer Institute, Frederick, MD, USA; Department of HIV and Sexual Health, King's College Hospital, London, UK; Department of Twin Research & Genetic Epidemiology, King's College London, London, UK; Department of Twin Research & Genetic Epidemiology, King's College London, London, UK

**Keywords:** APOL1, CKD, eGFR, epigenetics, nephropathy

## Abstract

**Background:**

Apolipoprotein L1 (APOL1) high-risk variants are major determinants of chronic kidney disease (CKD) in people of African ancestry. Previous studies have identified epigenetic changes in relation to kidney function and CKD, but not in individuals with *APOL1* high-risk genotypes. We conducted an epigenome-wide analysis of CKD and estimated glomerular filtration rate (eGFR) in in people of African ancestry and *APOL1* high-risk genotypes with HIV.

**Methods:**

DNA methylation profiles from peripheral blood mononuclear cells of 119 individuals with *APOL1* high-risk genotypes (mean age 48 years, 49% female, median CD4 count 515 cells/mm^3^, 90% HIV-1 RNA <200 copies/mL, 23% with CKD) were obtained by Illumina MethylationEPIC BeadChip. Differential methylation analysis of CKD considered technical and biological covariates. We also assessed associations with eGFR. Replication was pursued in three independent multi-ancestry cohorts with and without HIV.

**Results:**

DNA methylation levels at 14 regions were associated with CKD. The strongest signals were located in SCARB1, DNAJC5B and C4orf50. Seven of the 14 signals also associated with eGFR, and most showed evidence for a genetic basis. Four signals (in SCARB1, FRMD4A, CSRNP1 and RAB38) replicated in other cohorts, and 11 previously reported epigenetic signals for kidney function or CKD replicated in our cohort. We found no significant DNA methylation signals in, or near, the APOL1 promoter region.

**Conclusions:**

We report several novel as well as previously reported epigenetic associations with CKD and eGFR in individuals with HIV having *APOL1* high-risk genotypes. Further investigation of pathways linking DNA methylation to APOL1 nephropathies is warranted.

## Graphical Abstract

**Graphical Abstract:**
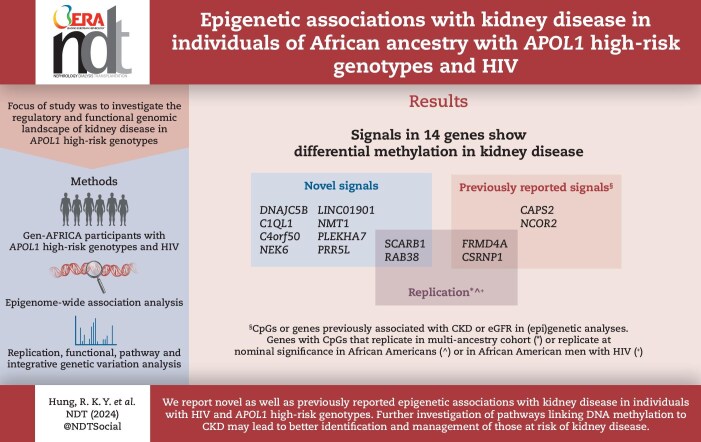

KEY LEARNING POINTS **What was known:**

- Chronic kidney disease (CKD) has multiple etiologies, where genetic variants in the *Apolipoprotein* L1 (*APOL1*) gene constitute major susceptibility risk.
- However, the regulatory and functional genomic landscape of CKD in the setting of *APOL1* renal risk genotypes is not well characterized.

**This study adds:**

1. We profiled epigenomic signatures of CKD in individuals with *APOL1* high-risk genotypes, identifying differentially methylated signals, with replication in external cohorts.
2. Our findings provide novel insights into the development of CKD in *APOL1* high-risk genotypes.

**Potential impact:**

- Further investigation into how these regulatory signals influence the development of kidney disease may lead to better identification and subsequent management of those at risk of kidney disease related to *APOL1.*

## INTRODUCTION

People of African ancestry are at substantially increased risk of chronic kidney disease (CKD), and this has partially been attributed to genetic variants of the *apolipoprotein* L1 (*APOL1*) gene [1, 2]. The risk of CKD is particularly high in those individuals with human immunodeficiency virus (HIV) [3]. The most common kidney diseases associated with genetic variants of the *APOL1*gene

are primary focal segmental glomerulosclerosis (FSGS), arterionephrosclerosis and collapsing glomerulopathies such as HIV-associated nephropathy (HIVAN) [4, 5]. The *APOL1* high-risk genotypes are homozygosity or compound heterozygosity for the G1 and G2 alleles (G1/G1, G2/G2 or G1/G2) [2], but how these variants differ mechanistically from the wild-type G0 allele remains unclear. An estimated 49% of end-stage kidney disease (ESKD) in people of African ancestry with HIV in the UK was attributable to *APOL1* high-risk genotypes [6]. However, about 70% of individuals with *APOL1* high-risk genotypes had normal kidney function [estimated glomerular filtration rate (eGFR) of ≥60 mL/min/1.73 m^2^], suggesting that interactions between genetic and environmental exposures contribute towards the development of CKD.

Epigenetic mechanisms such as DNA methylation are key regulators of gene expression that do not alter the underlying DNA sequence. DNA methylation modifications are stable and partially under genetic control [7], but can also be malleable in response to stochastic effects and environmental exposures. DNA methylation involves the addition of a methyl group to cytosine, and when this occurs in gene promoters it typically results in repression of gene expression. Recent epigenome-wide association studies (EWAS) have identified differentially methylated positions associated with eGFR and CKD [8, 9] in multi-ancestry samples and in American Africans [10]. Differentially methylated positions have also been associated with HIV viraemia, CKD, frailty, diabetes, liver function and mortality in people with HIV [11–16]. To date, only one EWAS of CKD has been conducted in men of African ancestry with HIV, and this study identified four differentially methylated positions associated with eGFR [17]. These studies provide evidence that DNA methylation may be a factor in the development or progression of CKD. Therefore, such analyses would be particularly relevant to individuals with HIV who have *APOL1* high-risk genotypes [4, 6, 18], in view of their exceptionally high risk of developing severe CKD.

The aim of this study was to investigate DNA methylation patterns in association with CKD and eGFR among participants with *APOL1* high-risk genotypes in the Genetic Markers of Kidney Disease Progression in People of African Ancestry with HIV (GEN-AFRICA) study [6].

## MATERIALS AND METHODS

### Study participants

The GEN-AFRICA study recruited individuals of self-reported African ethnicity living in the UK with HIV, aged 18 years and over, between May 2018 and February 2020. The present analyses are restricted to individuals with *APOL1* high-risk genotypes for whom longitudinal kidney function assessments from the time of HIV diagnosis [prior to starting antiretroviral therapy (ART)] were available. For the main analyses, we identified cases of CKD as confirmed *APOL1* nephropathy (HIVAN, FSGS or arterionephrosclerosis on kidney biopsy) or probable *APOL1* nephropathy (non-diabetic end-stage kidney disease and/or eGFR <60 mL/min/1.73 m^2^ with persistent heavy proteinuria [urine protein/creatine ratio (uPCR) >100 mg/mmol]. Controls had persistently normal kidney function (eGFR ≥60 mL/min/1.73 m^2^ and uPCR <30 mg/mmol) from HIV diagnosis. We also analyzed epigenetic associations with eGFR, using cross-sectional analysis of data assessments at the time of DNA sampling and using the creatinine-based CKD Epidemiology Collaboration equation without correction for ethnicity [19].

### Epigenomic data generation, processing and quality control

DNA methylation profiles were generated using the Illumina HumanMethylation EPIC array and *Enmix* [20] was used for methylation data processing, Quality control procedures are described in Supplementary data (see Supplementary Methods). DNA methylation blood cell subtype proportions for the study participants were estimated using the DNA Methylation Age Calculator [21] and based on the Houseman algorithm [22]. Epigenetic clocks were estimated using the DNA Methylation Age Calculator [21], and CKD epigenetic ageing association analysis focused on the original age acceleration residual estimates from the Horvath calculator and *GrimAge* acceleration estimates from the new methylation age calculator.

### Epigenome-wide association analysis

Mixed effect linear models (lme4 of *lmer* package in R) were used to assess associations between autosomal DNA methylation levels (response variable) and CKD status (predictor variable) (Supplementary data, Fig. S1). Fixed effects covariates included sex, age, smoking status, a history of diabetes and/or cardiovascular diseases, current CD4 cell count and nadir CD4 cell count (whole blood), proportion of granulocytes and monocytes, and methylation chip and position. To adjust for multiple comparisons, we applied a false discovery rate of 5%. The CKD methylation signals were additionally tested using eGFR as the predictor variable in sensitivity analyses.

### Replication

We sought to replicate our CKD DNA methylation signals in two independent datasets: Schlosser *et al*. [8], a multi-ancestry meta-analysis comprising 2376 CKD cases and 23 233 controls (not selected for HIV status), as well as in a subset of 4667 individuals of African-American ancestry within this cohort, and Chen *et al*. [17], a cohort of African-American with HIV comprising 96 CKD cases and 794 controls from the Veterans Aging Cohort Study (VACS) [23]. VACS is a prospective, observational cohort study of US military Veterans in the USA with and without HIV infection. Replication was deemed successful if effect estimates were in the same direction of association, and if the evidence for association surpassed Bonferroni correction for multiple testing (*P* = .05/8 for Schlosser *et al*. [8], and *P* = .05/14 for the Chen *et al*. [17] datasets). We also explored whether previously reported CKD and eGFR DNA methylation signals were associated with CKD in our cohort (see Supplementary Methods). We only considered CpGs identified from previous studies that also surpassed data quality control checks in our dataset, and were therefore available for testing (successfully tested) in our dataset.

### CpG annotations, functional and pathway analysis

We used CpG genomic annotations from the Illumina HumanMethylation EPIC manifest [24] to annotate CpGs to genes and genomic regions using hg19 genome reference. We also explored whether DNA methylation levels at the CKD-associated CpG sites identified in our study were previously associated with blood gene expression levels from the BIOS-BBMRI dataset [25]. To this end, we explored previously reported blood-based local expression Quantitative Trait Methylation (eQTM) results, defined as associations between DNA methylation levels and gene expression levels at each tested gene (“*cis-*eQTMs independent top effects” results from BIOS-BBMRI dataset). We also explored biological processes and molecular pathways, including Gene Ontology analysis, of the genes to which the most differentially methylated signals were annotated (see Supplementary Methods).

### Integrating DNA methylation signals with genetic variation and GWAS

We explored whether genetic variants influence DNA methylation levels at candidate CKD-associated CpG signals. To this end, we investigated if the most highly-associated CpGs were previously identified to harbor local (*cis*) methylation quantitative trait loci (mQTL) in samples from African-Americans from the Genetic Epidemiology Network of Arteriopathy (GENOA) study [26] and the repeated in samples of European ancestry, utilizing the Genetics of DNA Methylation Consortium (GoDMC) results [7] (see Supplementary Methods). Any identified single nucleotide polymorphisms (SNPs) that were previously identified as mQTLs for our most associated CpGs were further integrated with genome-wide association studies (GWAS) results to ascertain whether these were previously reported as GWAS signals associated with CKD.

### Ethics

The study was approved by an NHS Research Ethics Committee and Health Research Authority (18/LO/0234 and 239895). All participants provided written informed consent.

## RESULTS

A total of 119 participants with *APOL1* high-risk genotypes underwent DNA methylation profiling, consisting of 27 cases with CKD (14 biopsy-confirmed *APOL1* nephropathy: HIVAN 10, FSGS 3, hypertension 1), 55 controls with persistently normal kidney function and 37 individuals with previous acute kidney injury or mild CKD. The demographic and clinical characteristics of the 82 individuals who were cases with CKD and controls in the primary analyses are summarized in Table 1. Participants had a mean age of 47.5 [standard deviation (SD 8.6)] years and 49% were female. Most had long-standing HIV and were on ART with undetectable HIV-1 plasma RNA viral load. After quality control, a total of 767 414 unique CpG sites were included in downstream analyses.

**Table 1:** Baseline characteristics of study participants.

Participant characteristics	No CKD (*N* = 55)	CKD (*N* = 27)	Overall (*N* = 82)	*P*-value
Sex, female, *n* (%)	29 (52.7)	11 (40.7)	40 (48.8)	.40
Age, years, mean (SD)	46.9 (9.2)	48.8 (7.1)	47.5 (8.6)	.30
Region of ancestry, *n* (%)				
Caribbean	6 (10.9)	3 (11.1)	9 (11.0)	.99
Central Africa	1 (1.8)	0 (0)	1 (1.2)	
East Africa	1 (1.8)	1 (3.7)	2 (2.4)	
Southern Africa	2 (3.6)	2 (7.4)	4 (4.9)	
West Africa	42 (76.4)	21 (77.8)	63 (76.8)	
Other	3 (5.5)	0 (0)	3 (3.7)	
Time since HIV diagnosis, years, mean (SD)	13.2 (5.8)	13.7 (3.6)	13.4 (5.2)	.02
Nadir CD4 cell count, cells/mm^3^, median (IQR)	251 (133, 335)	65 (22, 148)	176 (73, 285)	<.0001
Current CD4 cell count, cells/mm^3^, median (IQR)	609 (475, 726)	413 (290, 580)	515 (405, 689)	.02
On antriretroviral therapy, *n* (%)	54 (98.2)	27 (100)	81 (98.8)	.78
HIV RNA <200 copies/mL, *n* (%)	51 (92.7)	23 (85.2)	74 (90.2)	.56
HBsAg positive, *n* (%)	5 (9.1)	3 (11.1)	8 (9.8)	1
Anti-HCV positive, *n* (%)	0 (0)	1 (3.7)	1 (1.2)	.36
Diabetes, *n* (%)	4 (7.3)	3 (11.1)	7 (8.5)	.90
Hypertension, *n* (%)	16 (29.1)	25 (92.6)	41 (50.0)	<.001
Cardiovascular disease^a^, *n* (%)	1 (1.8)	3 (11.1)	4 (4.9)	.19
Smoking status, *n* (%)				.90
Never	47 (85.5)	22 (81.5)	69 (84.1)	
Current/past smoker	8 (14.5)	5 (18.5)	13 (15.9)	
eGFR,^b^ mL/min/1.73 m^2^, *n* (%)				<.0001
≥90	35 (63.6)	1 (3.7)	36 (43.9)	
60–89	20 (36.4)	1 (3.7)	21 (25.6)	
30–59	0 (0)	4 (14.8)	4 (4.9)	
15–29	0 (0)	2 (7.4)	2 (2.4)	
<15 (ESKD^c^)	0 (0)	19 (70.4)	19 (23.2)	

HBsAg, hepatitis B surface antigen; HCV, hepatitis C virus; IQR, interquartile range; CKD-EPI, Chronic Kidney Disease Epidemiology Collaboration.

a Cardiovascular disease = composite of any previous history of myocardial infarction, coronary artery disease, peripheral vascular disease, stroke, heart failure and cardiomyopathy.

b eGFR calculated with 2021 CKD-EPI formula without correction for ethnicity.

c ESKD = eGFR <15 mL/min/1.73 m^2^.

### Differential methylation signatures of CKD in APOL1 high-risk genotypes

Epigenome-wide association analyses identified 14 CpG sites that were significantly associated with CKD (Table 2). The four most associated CpG signals were cg14849578 located in *SCARB1*, cg04725636 in *DNAJC5B*, cg06368300 near *C1QL1*, and cg18633191 in *C4orf50*. The 14 identified CpGs were located in 10 genes. Three CpG sites were in gene promotor regions, including cg13958199, cg15716304 and cg05348871 located 1.5 kb from the transcription start site of *NEK6, CAPS2* and *PRR5L*, respectively. Eight CpG sites were located within genes, including cg04725636, cg24791666 and cg01936957 at the 5′ untranslated region (UTR) of *DNAJC5B, CSRNP1* and *NCOR2*, respectively; and the most strongly associated signal, cg14849578, at the 3′UTR of *SCARB1*. Four CpG sites, cg18633191, cg22959742, cg06720945 and cg09263059, were located in introns of *C4orf50, FRMD4A, PLEKHA7* and *NMT1*, respectively. Finally, three CpG sites were not in or near genes, and these included cg06368300, located 20 kb from *C1QL1*, cg05245822, located 45 kb from *LINCO1901* and cg12769599 located 10 kb from *RAB38* (Table 2). None of the CpG sites located in the *APOL1* gene or the *APOL1* promoter region was associated with CKD (Supplementary data, Table S1).

**Table 2:** Epigenome-wide associated CKD-associated CpGs (FDR <0.05) discovery, sensitivity and replication results.

				Discovery cohort				Replication			
				CKD (primary outcome)		eGFR (sensitivity)		Schlosser *et al*.^b^		Chen *et al*.^c^	
Cgs	Chrom Pos	Nearest gene	Location	Effect (SE)	*P*-value (FDR)	Effect (SE)	*P*-value (FDR)	Effect (SE)	*P*-value	Effect (SE)	*P*-value
cg14849578^a^	12	*SCARB1*	3′UTR	0.069 (0.012)	5.94E-09 (0.003)	–0.005 (0.004)	8.55E-07 (0.017)	0.003 (0.001)	2.17E-03	0.009 (0.004)	.026
cg04725636	8	*DNAJC5B*	5′UTR	–0.157 (0.028)	1.03E-08 (0.003)	0.010 (1.004)	1.26E-07 (0.007)	NA	NA	0.012 (0.012)	.292
cg06368300	17	*C1QL1*	20 kb	0.086 (0.016)	3.14E-08 (0.007)	–0.005 (0.005)	1.87E-03 (0.254)	–0.002 (0.002)	2.92E-01	–0.004 (0.007)	.509
cg18633191	4	*C4orf50*	4 kb	0.074 (0.013)	4.38E-08 (0.007)	–0.013 (0.004)	3.00E-08 (0.003)	NA	NA	0.011 (0.008)	.171
cg13958199	9	*NEK6*	TSS1500	0.053 (0.010)	8.24E-08 (0.01)	–0.006 (0.003)	2.13E-05 (0.057)	0.001 (0.001)	4.73E-02	0.000 (0.003)	.992
cg05245822	18	*LINC01901*	45 kb	–0.132 (0.024)	9.37E-08 (0.01)	0.009 (0.005)	1.03E-06 (0.018)	NA	NA	–0.024 (0.014)	.091
cg22959742^a^	10	*FRMD4A*	Intron	0.092 (0.019)	3.68E-07 (0.024)	–0.005 (0.003)	1.27E-05 (0.048)	0.006 (0.001)	6.04E-08	0.003 (0.006)	.653
cg15716304	3	*CAPS2*	TSS1500	–0.011 (0.002)	3.81E-07 (0.024)	0.009 (0.005)	1.01E-02 (0.412)	NA	NA	–0.012 (0.007)	.074
cg09263059	17	*NMT1*	Intron	0.067 (0.014)	3.94E-07 (0.024)	–0.002 (0.004)	6.16E-03 (0.362)	–0.001 (0.001)	5.74E-01	0.002 (0.005)	.645
cg24791666^a^	3	*CSRNP1*	5′UTR	0.038 (0.008)	4.44E-07 (0.024)	–0.003 (0.003)	3.31E-04 (0.145)	0.002 (0.001)	3.59E-03	0.001 (0.002)	.693
cg06720945	11	*PLEKHA7*	Intron	0.068 (0.013)	4.48E-07 (0.024)	–0.005 (0.002)	8.66E-07 (0.017)	NA	NA	0.008 (0.006)	.176
cg12769599^a^	11	*RAB38*	10 kb	–0.060 (0.013)	4.59E-07 (0.024)	0.004 (0.005)	1.86E-04 (0.119)	–0.004 (0.001)	1.24E-03	–0.007 (0.007)	.320
cg05348871	11	*PRR5L*	TSS1500	–0.087 (0.021)	7.22E-07 (0.035)	0.001 (0.003)	1.55E-03 (0.238)	–0.002 (0.001)	1.93E-01	–0.004 (0.006)	.426
cg01936957	12	*NCOR2*	5′UTR	0.044 (0.010)	8.33E-07 (0.038)	–0.006 (0.004)	7.90E-06 (0.037)	NA	NA	0.003 (0.004)	.456

^a^CpGs that met replication threshold.

^b^Multi-ancestry cohort.

^c^The VACS were African-Americans with HIV.

We considered the effect size of the 14 reported DNA methylation signals by calculating the mean difference in DNA methylation levels between individuals with and without CKD (Table S2). We observed that the difference in (unadjusted) DNA methylation levels between participants with CKD and without CKD were typically modest, <4.5%, apart from two signals at cg04725636 located in *DNAJC5B* (mean difference = –10.2%) and cg05245822 located in *LINCO1901* (mean difference = –12.8%).

Seven of the 14 CKD-associated CpG sites showed nominally significant associations (*P* < .05) with eGFR, of which one surpassed multiple testing correction (cg18633191 located in *C4orf50, P* < .004). Six of the nominally associated CpG sites were located within *SCARB1, DNAJC5B, LINCO1901, FRMD4A, PLEKHA7* and *NCOR2*. The direction of methylation association was consistent with the discovery analyses (e.g. hypermethylation was associated with both CKD and lower eGFR).

### Replication of CKD-methylation associations

We sought to replicate the 14 CKD-associated methylation signals in two external datasets. In the multi-ancestry dataset comprising individuals of unknown HIV status (2376 CKD cases and 23 233 controls) [8], we replicated four of eight tested CKD-associated methylation signals (at Bonferroni correction, *P* < .006; Table 2 and Supplementary Table S3)—cg14849578 in *SCARB1*, cg22959742 in *FRMD4A*, cg24791666 in *CSRNP1* and cg12769599 near *RAB38*. When these analyses were restricted to African-Americans (404 CKD cases and 4263 controls), these CpG sites remained concordant in direction of association, with three signals showing nominally significant associations (*P* < .05; cg14849578 (*SCARB1*), cg22959742 (*FRMD4A*), cg24791666 (*CSRNP1*)).

We also sought replication of our DNA methylation signals in a cohort of African-American men with HIV in the VACS (96 CKD cases and 794 controls) [17]. All 14 CKD-associated methylation signals were tested, 12 of which showed consistent direction of association and one was nominally significant (cg14849578 in *SCARB1*; Table 2, Supplementary data, Table S3).

### Evaluation of previously reported methylation signals of CKD and eGFR

Replication was also pursued in our dataset for CKD-associated CpG sites identified in previously published epigenome-wide analyses. Of the 53 CpGs sites identified in the multi ancestry cohort [8], we tested 44 and found 6 (11%) to be associated with CKD after multiple testing correction, with the same direction of association (Table S4). The replicated sites were cg00501876 (*CSRNP1*), cg02304370 (*PHRF1*), cg04578362 (*TEX41*), cg06158227 (*ZSCAN29*), cg14029001 (*CCND3*) and cg17944885 (*ZNF788*).

We additionally pursued replication of three sets of previously reported eGFR-associated CpGs in our full cohort (Supplementary data, Table S5). We successfully replicated 9 (16%) of 58 tested eGFR-associated CpGs in the multi-ancestry cohort by Schlosser *et al*. [8], and three (5%) of 65 tested CpGs in the multi-ancestry cohort by Breeze *et al*. [9] (Supplementary data, Table S5). When the Breeze *et al*. [9] dataset was restricted to African-Americans, we successfully replicated all three signals tested of 23 reported CpGs. Finally, we replicated one of four tested CpGs reported by Chen *et al*. [17] (cg17944885); the direction of methylation was consistent across all four tested CpG sites. Of these signals, cg17944885 located in *ZNF20* (previously reported in *ZNF788*) was replicated and reported to be associated with both CKD and eGFR across all studies considered [effect estimate for CKD in our cohort = –0.013, standard error (SE) = 0.003, *P* = 1.26 × 10^−5^].

### Functional exploration of CKD-associated methylation signals

We first explored whether our CKD methylation signals had putative effects on gene function. To this end, we assessed whether DNA methylation levels at the 14 peak CpG sites were correlated with the nearest gene's expression levels in previously published methylation–expression correlation results [27, 28], and found that cg14849578 (located in *SCARB1*) was negatively correlated to *SCARB1* gene expression (Supplementary data, Table S6).

We next explored potential functional impacts of the CKD signals. At a more relaxed and exploratory threshold of false-discovery rate (FDR) 20%, there were 504 CpGs annotating to 334 unique genes. Gene Ontology investigation indicated that these genes are involved in 7 biological processes (Supplementary data, Table S7A) and 12 molecular function pathways (Supplementary data, Table S7B, see Supplementary Results). Pathway analysis of the 334 genes identified the top results (Supplementary data, Table S8) to be related to cellular signal transduction, immune system signaling pathways, including involvement in metabolic and infectious disease [29].

Finally, we explored whether our CKD signals (at FDR 20%) overlapped with loci from previously published genetic or epigenetic associations with relevant phenotypes and diseases. Comparison of these signals with previous GWAS (2023 GWAS and the UK Biobank GWAS catalogs) showed that some of our loci overlap GWAS regions associated with traits related to CKD and hypertension (Supplementary data, Tables S9 and S10). We then investigated whether any of the 504 CKD associated CpGs and 334 genes at FDR 20% were previously reported to be associated with kidney function or kidney disease at epigenome-wide level. At the CpG level, cg22959742, cg24791666 and cg01936957 located in the *FRMD4A, CSRNP1* and *NCOR2* genes, respectively, were reported to be associated with CKD (Supplementary data, Table S11). At the gene level three genes (*SLC15A4, TXNIP* and *IGF2BP2*) were previously reported to be associated with systolic and diastolic blood pressure [30, 31] (Supplementary data, Table S12A) and 39 of our 42 genes were reported to be associated with CKD by Chu *et al*. [32] and Hillary *et al*. [33] (Supplementary data, Table S12B). Overall, *NCOR2* and *FRMD4A* were consistently reported to be associated with kidney disease when referenced against the GWAS and EWAS catalogs.

### Genetic basis of CKD-associated methylations

To explore whether genetic variation may contribute to our CKD signals, we integrated our CKD-methylation results with previously reported DNA methylation Quantitative Trait Loci (mQTLs) obtained from African-Americans [26]. We found that 13 of 14 CKD-associated CpGs were under local genetic control or harbored *cis*-mQTLs (Supplementary data, Table S13A). Referencing against individuals of European ancestry in the GoDMC study, we observed a similar trend where seven CpGs had evidence for *cis-*mQTLs [7]. The single CpG (cg09263059, located in the intron of *NMT1*) that had no mQTLs in those of African ancestry was found to harbor *cis-*mQTLs in those of European ancestry. While some genes that the mQTL SNPs from the GENOA cohort were annotated to were also the genes that our top 14 CpG signals annotated to (*DNAJC5B, LRRK2, C1QL1, NEK6, FRMD4A, CAPS, CSRNP1, PLEKHA7, RAB38, NCOR2*), other SNPs annotated to a further seven unique genes (*GORASP1, TTC21A, XIRP1, TRIM55, GLIPR1L1, K1F18B* and *DCAKD*). Of these, *DCAKD* was previously reported to be associated with systolic blood pressure [34] (Supplementary data, Table S14).

Most of the previously reported EWAS CKD-associated CpG-sites that we replicated were also found to be influenced by genetic variation. For example, all six replicated CpGs from the multi-ancestry cohort by Schlosser *et al*. [8] had *cis-*mQTLs in the GoDMC study, and four had *cis-*meQTLs in the GENOA database (Supplementary data, Table 13B).

### Accelerated epigenetic ageing in CKD

Beyond single CpG analyses, we also considered whether summary measure epigenetic variables showed associations with CKD. *GrimAge* of our 119 study participants exceeded chronological age by 17.8 (mean, SD 5.1) years, with significant evidence for *GrimAge* acceleration among individuals with CKD (adjusted Beta = 2.91, SE = 1.02, *P* ≤ .001). Conversely, there was no evidence for age acceleration in individuals with CKD when the original Horvath DNA methylation age clock was used (adjusted beta = 3.44. SE = 1.99, *P* = .104).

## DISCUSSION

In this African diaspora cohort of people with HIV, *APOL1* high-risk variants and well-defined kidney phenotypes, we identified 14 CpG sites in 10 genes that were differentially methylated in those with CKD when compared with those with persistently normal kidney function. We successfully replicated four of these CpGs in other cohorts, and we also replicated 11 previously published methylation signals linked to CKD or eGFR in our study participants.

The most strongly associated CKD-associated CpG (cg14849578) was located in 3′UTR region [a region that regulates mRNA translation of the gene encoding scavenger receptor class B type 1 (*SCARB1*)]. SCARB1 is involved in reverse cholesterol transport and high-density lipoprotein (HDL) homeostasis and plays a role in reducing intracellular inflammation and oxidation [35]. *SCARB1* expression in macrophages and endothelial cells modifies cholesterol trafficking in atherosclerotic lesions by limiting foam cell formation and subsequent inflammation [36, 37]. Several studies have reported that loss of *SCARB1* function is associated with increased cardiovascular disease risk [38, 39]. There is growing evidence that impaired HDL function is a contributing factor to kidney damage and disease progression [40–42]. Recently, a study reported that *SCARB1* dysfunction in mice led to development of spontaneous intraglomerular lipoprotein deposits which may point towards an increased risk of developing metabolic nephropathy in *SCARB1* loss-of-function variants [43]. In our study, the *SCARB1* signal (cg14849578) was found to be hypermethylated in those with kidney disease including *APOL1* nephropathy, and results from the BIOS-BBMRI dataset show that this methylation signal was negatively associated with *SCARB1* gene expression. Taken together, these two sets of results suggest that *SCARB1* expression may be downregulated in those with kidney disease in *APOL1* high-risk genotypes. Cg14849578 was also reported to be associated with HIV [44] and it is possible that *SCARB1* interacts with immunodeficiency and HIV or share a common pathway leading to CKD.

Differential methylation of CpG sites in *CSRNP1* and *FRMD4A* was also successfully replicated. The CpG sites in *CSRNP1* are located in the 5′UTR region, a region critical for gene translation, while the CpG site for *FRMD4* was located in the intron where it may affect transcriptional regulation. Expression of *CSRNP1* has been reported to be induced by Interleukin-2 in mouse models [45] and upregulated *CSRNP1* can impede cell cycle progression and induce cell apoptosis via the c-jun N-terminal kinase (JNK) of the mitogen-activated protein kinase (MAPK) signaling pathways in *Drosophila* [46]. The resulting apoptosis may activate Janus kinase/signal transducer and activator of transcription (JAK/STAT) pathway [47]. The JAK/STAT pathway can also be activated by pro-inflammatory cytokines and high interferon states [48, 49]. Activation of the JAK/STAT pathway is a well-recognized step in several forms kidney disease including diabetic nephropathy [50, 51], autosomal dominant polycystic kidney disease [52], HIVAN [53] and COVID-associated glomerulopathy [54]. Indeed, tetracycline-induced *APOL1* G1 or G2 expression in kidney cells resulted in activation of the JNK pathway [55] and these results were reflected in a more recent study where interferon induced *APOL1* G1 expression in mouse models was reduced by a JAK1/2 inhibitor [56]. Besides that, Mendelian randomization analysis of cg00501876 (located within/near *CSRNP1*) also suggests a causal role in eGFR decline [8]. *FRMD4A* is involved in protein scaffolding that regulates epithelial cell polarity. Its function in kidney disease is currently unknown and may represent a novel pathway of CKD in people with HIV and *APOL1* high-risk genotypes.

When referenced against published findings in the GWAS and EWAS catalog, *NCOR2*, a nuclear receptor coregulator, has repeatedly been associated with kidney disease. Several CpG sites in the 5′UTR region of *NCOR2* were differentially methylated with CKD in our cohort, both genome-wide (FDR 5%) and at more relaxed thresholds (FDR 20%). Nuclear receptors are master regulators of lipid and carbohydrate metabolism and play a pathogenic role in renal lipid accumulation [57]. *NCOR2* is also closely involved in apoptosis, cell survival and cycle progression [58, 59], and linked to inflammation [60]. As *NCOR2* is involved in both renal lipid metabolism and inflammation, it is possible that regulation of this gene presents a novel pathway to development of *APOL1* nephropathy in those with HIV.

Strikingly, most of the top 14 differential methylation signals in our study appear to be influenced by genetic variants located near to these sites, suggesting that there is genetic variation between those with and without CKD in *APOL1* high-risk genotypes. Indeed, the *APOL1* p.N264K missense variant was recently reported to mitigate the increased risk of CKD and ESKD in *APOL1* high-risk genotypes [61] and also protected against G2-associated FSGS [62]. Difference in genetic variants may drive predisposition to DNA (de)methylation and influence the maintenance or deletion of methylation marks and these changes in methylation may then influence gene expression leading to or protecting against disease. A recent study by Breeze *et al*. [63] in *APOL1* high-risk genotypes reported four out of five CpGs showing significant meQTLs but these four CpGs were not associated with CKD in our cohort.

We observed evidence for accelerated epigenetic ageing in individuals with CKD in our cohort, based on *GrimAge* acceleration estimates, which are strongly linked to mortality [64]. Infection with HIV has also been associated with epigenetic ageing [21, 65], which can be mitigated by the use of ART [66]. Similarly, CKD has also previously been associated with accelerated epigenetic ageing up to 3 years [67, 68]. The epigenetic age of our participants with *APOL1* nephropathy who generally had well controlled HIV is in keeping with these findings.

Identification of DNA methylation signals associated with CKD is an important first step towards defining epigenetic mechanisms that may contribute to the development or progression of kidney disease in this population. Our study has several limitations, including the relatively small sample size, potential for inflation bias, use of DNA from peripheral blood mononuclear cells rather than kidney cells, and the cross-sectional study design where DNA methylation may vary over time and be a cause or consequence of CKD. Similar trait-associated CpG methylation findings in blood and target tissues have previously been reported, which mitigates the use of peripheral blood for DNA methylation analyses [32]. We successfully replicated some of our signals in other cohorts and replicate previously published CpGs associated with kidney disease and function [69] further validating our results. When replication of our results was restricted to African-Americans with HIV, the lack of significant association, but consistent directionality of effect, could be due to several factors. These include limited statistical power for CKD consistent with *APOL1* nephropathy in the replication cohort, differences in the severity of CKD, differences in confounding factors, or a combination of these effects. Further EWAS studies with larger sample sizes in those with *APOL1* high-risk genotypes, as well as use of DNA methylation derived from kidney tissue will improve our understanding of the regulatory mechanisms of CKD in *APOL1* high-risk genotypes and hopefully shed more light on the pathophysiological mechanisms of *APOL1* nephropathy. We are also unable to account for psychosocial or other environmental or systemic factors that could be influencing the rate of epigenetic ageing in our cohort. However, we report similar ageing rates to previously published reports, thus making epigenetic ageing in our cohort less likely to be affected by the aforementioned factors. Finally, viral entities that exist in those with chronic HIV such as HIV clades, co-infections with cytomegalovirus and Epstein–Barr virus, and exposure to immunosuppressive medications in kidney transplant recipients are potential confounders that we are unable to account for.

In summary, we report both novel and previously identified epigenetic associations with CKD in individuals with HIV. DNA methylation signals in *SCARB1, FRMD4* and *CSRNP1* are associated with CKD across all ancestries and may represent a unique mechanism for HIV-related CKD. Most of the DNA methylation sites associated with CKD in our cohort were under genetic control, supporting the hypothesis that there are other genes that influence *APOL1* variant expression. Further investigation examining the pathways linking DNA variants, DNA methylation and *APOL1* variant expression to CKD in *APOL1* high-risk genotypes, within and outside of the setting of HIV, is likely to provide better insights into the development of kidney disease in this patient population.

## Supplementary Material

gfae237_Supplemental_File

## Data Availability

The informed consent given by the study participants does not cover data deposit in public repositories. Access to the study data and samples is governed by the National Health Service data access policy and those of King's College Hospital NHS Foundation Trust, the study sponsor. The Gen-AFRICA cohort is open to collaborations, and all requests from researchers who meet the criteria for access to fully anonymized patient level data will be considered. Concepts can be submitted for review to the principal investigator (F.A.P., E-mail: frank.post@kcl.ac.uk).
