# Supplementary material for: Epigenetic associations with kidney disease in individuals of African ancestry with APOL1 high-risk genotypes and HIV
Source: Nephrol Dial Transplant. 2024 Oct 24;40(5):997–1006. doi: 10.1093/ndt/gfae237 (PMC12035534; doi:10.1093/ndt/gfae237)
Supplement: gfae237_Supplemental_File [file gfae237_supplemental_file.pdf]

Supplementary Methods

Epigenomic Data Generation, Processing and Quality Control

DNA was extracted from peripheral blood buffy coats which had been stored at -70° until use. Enmix (1) was used for methylation data processing and quality control including background and dye bias correction, quantile normalization of signals and estimation of adjusted beta-values. CpG signals with detP > 0.000001 and Nbead < 3 were assigned as missing data. Minfi (2) was then used to exclude samples with median methylated and unmethylated signals below 10.5. Exclusions based on missing data were set for CpGs filtered at 5%, and for samples filtered at 1%. Further CpGs were excluded based on previously described cross-reactive/non-specific probes (3) (4) and multimapping probes that mapped to the hg19 genome reference with up to 2 mismatches using Bowtie (5). To account for whole blood cell heterogeneity, the estimated of 10 blood cell types (monocytes, granulocytes, natural killer cells, naive CD8 and CD4 cells (6), CD8 and CD4 T cells, memory and effector cells [CD8pCD28nCD45Ran], plasmablasts and B cells) was done using the Houseman et al (7) algorithm, implemented in the online Horvath calculator (8).

Epigenome-wide association analyses

Prior to analysis DNA methylation values for each CpG site were normalized to N(0,1).Effect sizes estimates were obtained by fitting the same linear mixed effects model, but without normalizing DNA methylation values to N(0,1). Quality assessment of the epigenome-wide association test statistics included estimating lambda, the inflation factor for each EWAS. Where the global inflation factor was over 1 (lambda = 1.304 for main analysis [Figure S2]), we applied the *Bacon* R package (9) to the EWAS results. Multiple testing adjustment was carried out by applying a false discovery rate of 5% using the 'fdr' function from the *lmer* R package.

Finally, DNA methylation age of the study participants was estimated using the Horvath's DNA Methylation Age Calculator (8), and analyses focused on *GrimAge* (10). Linear regression models were fitted with *AgeAccelGrim* as the response variable and CKD status (*APOL1* nephropathy vs no CKD) as predictor variable, with further adjustments for the same covariates as the primary epigenome-wide analyses.

Replication Cohorts

We investigated if our top CKD-associated methylation signals were found in previously reported DNA methylation signals for CKD and eGFR from three published studies, including Schlosser et al (11) (CKD and eGFR), Chen et al. (6) (eGFR), and Breeze et al (12) (eGFR) in multi-ancestry population samples (Table S3, S5 and S5). Schlosser et al (11) investigated DNA methylation in whole blood of 33,605 individuals across 36 population studies and reported 69 CpGs associated with eGFR, of which 53 were also associated with prevalent CKD. Chen et al (6) reported 4 CpGs associated with eGFR in African Americans with HIV, and Breeze et al (12) identified 78 eGFR-associated CpGs in 5,428 participants, including 23 CpGs when analyses were restricted to African Americans. Replication of previously reported methylation associations with kidney disease or kidney function was deemed successful if the strength of association surpassed multiple testing correction, with the same direction of effect.

CpG annotations, functional and pathway analysis

A more relaxed FDR cutoff of 20% was applied for pathway analysis, where CKD-associated CpGs that met that threshold were analysed in EnrichR (13) utilizing Illumina EPIC genes as the background gene set. We then explored if previously published studies had reported associations between genetic variants in the genes annotated to our candidate signals and any diseases or phenotypes in the 2023 GWAS catalog (14) and UK Biobank GWAS catalog (15). Finally, we interrogated the EWAS catalog (16) to assess if the most associated signals were previously published in context of kidney disease outcomes (including hypertension, albuminuria, proteinuria, eGFR).

Integrating DNA methylation signals with genetic variation and GWAS

We explored if genetic variants influence DNA methylation levels at candidate CKD-associated CpG signals. To this end, we investigated if the most highly-associated CpGs were previously identified to harbour local (*cis*) methylation quantitative trait loci (mQTL) in samples from African

Americans from the Genetic Epidemiology Network of Arteriopathy (GENOA) study (17) and the repeated in samples of European ancestry, utilizing the Genetics of DNA Methylation Consortium (GoDMC) results

Integrating DNA methylation signals with genetic variation and GWAS

To investigate if the CKD-associated CpGs was previously identified to harbour cis mQTLs in the GENOA and GoDMC samples, we considered local genetic effects, exploring all single nucleotide polymorphisms (SNPs) that were within 50kb of the 14 CKD-associated CpGs. This allowed us to assess if there was a difference in genetic variants associated with DNA methylation levels at candidate CKD-associated CpG signals between ancestries.

Supplementary Results

Functional exploration of APOL1 nephropathy-associated methylation signals

At a more relaxed threshold of FDR 20%, there were 504 CKD-associated CpGs annotating to 334 unique genes in our cohort. Gene ontology investigation indicated that these genes are involved in seven biological processes (Table S7A) including organelle organization, regulation of DNA-template and RNA Polymerase II transcription, monoacylglycerol metabolic and catabolic process, cellular response to organic substances and protein-containing complex assembly. These 334 genes also annotated to 12 molecular function pathways including cis-regulatory DNA binding that may suggest that DNA methylation is disrupting binding sites. Signaling adaptor activities, mRNA, protein, and lipid binding activities at a molecular level were also indicated (Table S7B).

Figure S1: Flowchart of EWAS analysis

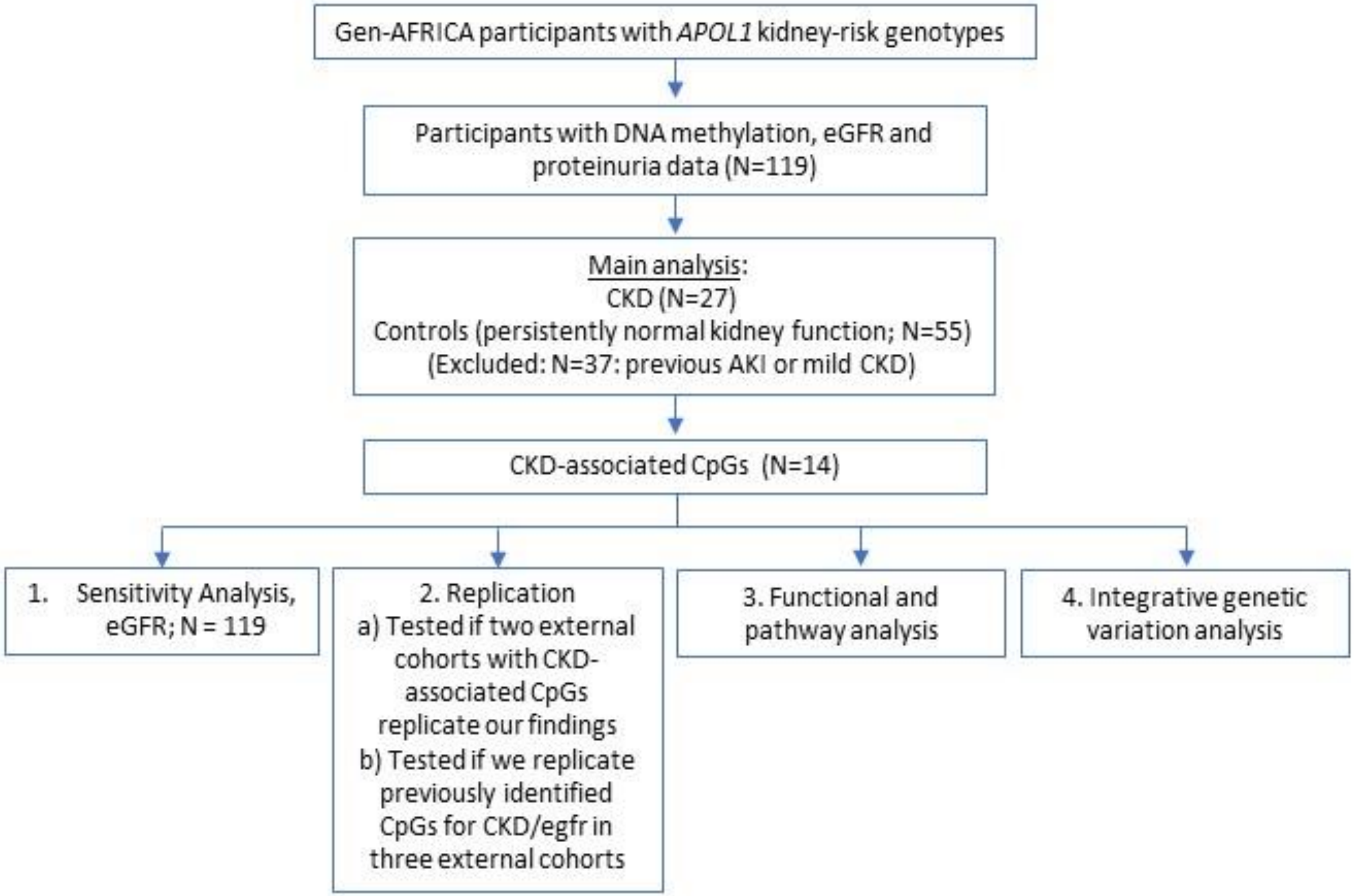

Figure S2: QQ plot of CKD vs no CKD

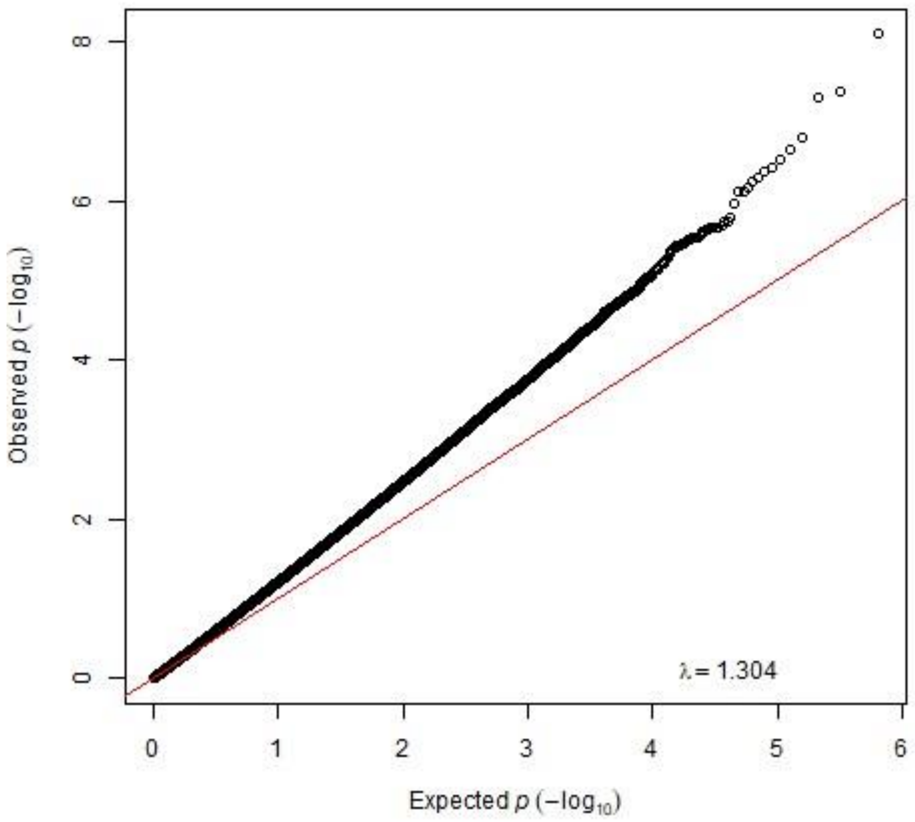

Supplementary Table 1: CpGs located within, or within 1kb from the start of the *APOL1* gene

Cgs*	Effect estimate	Standard Error	p value	FDR
cg08415592	-0.011	0.012	0.620	1
cg18448426	-0.020	0.008	0.087	1
cg11804381	-0.003	0.003	0.544	1
cg03715078	-0.011	0.016	0.652	1

*CpGs that were successfully tested in our participants

Supplementary Table 2: Difference in unadjusted methylation levels between participants with CKD and those with no CKD.

Cgs	Nearest gene	No CKD		CKD		% difference
		Mean	SD	Mean	SD	
cg14849578	*SCARB1*	0.755	0.055	0.773	0.06	1.8
cg04725636	*DNAJC5B*	0.535	0.092	0.433	0.128	-10.2
cg06368300	*C1QL1*	0.303	0.056	0.341	0.069	3.8
cg18633191	*C4orf50*	0.225	0.058	0.256	0.052	3.1
cg13958199	*NEK6*	0.674	0.059	0.669	0.052	-0.5
cg05245822	*LINC01901*	0.835	0.06	0.707	0.105	-12.8
cg22959742	*FRMD4A*	0.593	0.094	0.606	0.08	1.3
cg15716304	*CAPS2*	0.954	0.007	0.945	0.009	-0.9
cg09263059	*NMT1*	0.52	0.059	0.543	0.045	2.3
cg24791666	*CSRNP1*	0.532	0.047	0.522	0.042	-1
cg06720945	*PLEKHA7*	0.504	0.087	0.474	0.103	-3
cg12769599	*RAB38*	0.098	0.049	0.054	0.029	-4.4
cg05348871	*PRR5L*	0.355	0.128	0.387	0.131	3.2
cg01936957	*NCOR2*	0.561	0.048	0.562	0.035	0.1

Supplementary Table 3: Replication of other datasets/cohorts to current study (CKD)

**Discovery**							**Schlosser et al^**						**Chen et al***		
CKD CpGs	Chrom pos	Nearest gene	Location/ distance from gene	Effect estimate	Standard error (SE)	P value	Multi-ancestry effect estimate	Multi-ancestry SE	Multi-ancestry P value	AA effect estimate	AA SE	AA P value	Effect estimate	SE	P-value
cg14849578	12	*SCARB1*	3'UTR	0.069	0.012	5.94E-09	0.003	0.001	2.17E-03	0.005	0.003	4.57E-02	0.009	0.004	2.58E-02
cg04725636	8	*DNAJC5B*	5'UTR	-0.150	0.028	1.03E-08							0.012	0.012	2.92E-01
cg06368300	17	*C1QL1*	20kb	0.086	0.016	3.14E-08	-0.002	0.002	2.92E-01	0.002	0.004	6.25E-01	-0.004	0.007	5.09E-01
cg18633191	4	*C4orf50*	4kb	0.074	0.013	4.38E-08							0.011	0.008	1.71E-01
cg13958199	9	*NEK6*	TSS1500	0.053	0.010	8.24E-08	0.001	0.001	4.73E-02	0.002	0.002	1.67E-01	0.000	0.003	9.92E-01
cg05245822	18	*LINC01901*	45kb	-0.130	0.024	9.37E-08							-0.024	0.014	9.08E-02
cg22959742	10	*FRMD4A*	Intron	0.092	0.019	3.68E-07	0.006	0.001	6.04E-08	0.008	0.003	1.46E-02	0.003	0.006	6.53E-01
cg15716304	3	*CAPS2*	TSS1500	-0.010	0.002	3.81E-07							-0.012	0.007	7.35E-02
cg09263059	17	*NMT1*	Intron	0.067	0.014	3.94E-07	-0.001	0.001	5.74E-01	0.002	0.004	4.82E-01	0.002	0.005	6.45E-01
cg24791666	3	*CSRNP1*	5'UTR	0.038	0.008	4.44E-07	0.002	0.001	3.59E-03	0.004	0.001	1.20E-02	0.001	0.002	6.93E-01
cg06720945	11	*PLEKHA7*	Intron	0.068	0.013	4.48E-07							0.008	0.006	1.76E-01
cg12769599	11	*RAB38*	10kb	-0.060	0.013	4.59E-07	-0.004	0.001	1.24E-03	-0.005	0.004	1.55E-01	-0.007	0.007	3.20E-01
cg05348871	11	*PRR5L*	TSS1500	-0.080	0.021	7.22E-07	-0.002	0.001	1.93E-01	-0.004	0.003	2.00E-01	-0.004	0.006	4.26E-01
cg01936957	12	*NCOR2*	5'UTR	0.044	0.010	8.33E-07							0.003	0.004	4.56E-01

AA = African Americans;

^Bonferroni p value = 0.006 (0.05/8)

* The VACS cohort used were HIV+ African Americans, Bonferroni p value = 0.0004 (0.05/14)

Supplementary Table 4A: Replication of previously published CpGs associated with CKD: Summary Table

Study	No of CpGs associated with CKD	No of CpGs successfully tested in our participants	Bonferroni p value*	No of CpGs replicated^	% replicated
Schlosser (multi-ancestry)	53	44	1.14E-03	6	11

* Bonferroni p value = 0.05/no of CpGs successfully tested

^CpGs that meet the Bonferroni p value

Supplementary Table 4B: Replication of previously published CpGs associated with CKD: Full results

	Schlosser et al				Discovery		
CKD CpGs	Nearest Gene	Effect size	SE	p value	Effect size	SE	p value
cg00501876	*CSRNP1*	3.52E-03	6.94E-04	3.92E-07	0.033	0.009	6.56E-04
cg02304370	*PHRF1*	-8.11E-03	1.02E-03	1.51E-15	-0.071	0.018	2.01E-04
cg04578362	*TEX41*	4.26E-03	8.64E-04	8.25E-07	0.042	0.012	1.16E-04
cg06158227	*ZSCAN29*	4.02E-03	8.15E-04	8.00E-07	0.024	0.006	1.14E-04
cg14029001	*CCND3*	3.83E-03	7.60E-04	4.89E-07	0.023	0.007	4.46E-04
cg17944885	*ZNF788*	7.55E-03	7.29E-04	3.68E-25	0.018	0.005	1.62E-06

Supplementary Table 5A: Replication of previously published CpGs associated with eGFR: Summary table

Studies	No of CpGs associated with eGFR	No of CpGs successfully tested in our participants	Bonferroni p value*	No of CpGs replicated^	% replicated
Schlosser (multi-ancestry)	69	58	8.62E-04	9	16
Breeze (multi-ancestry)	78	65	7.69E-04	3	5
Breeze (African Americans only)	23	3	1.67E-02	3	100
Chen 2023 (VACS HIV AA)	4	4	1.25E-02	1	25

* Bonferroni p value = 0.05/no of CpGs successfully tested
^CpGs that meet the Bonferroni p value

Supplementary Table 5B: Replication of previously published CpGs associated with eGFR: Individual studies with results

Breeze et al (Multi ancestry)						Discovery		
CpG associated with eGFR	Chr	Position (hg38)	Gene	Effect estimate	p value	Effect estimate	SE	p value
cg04682977	7	1023075	C7orf50;MIR339	-15.0496	0.00110	-0.0091	0.0026	4.20E-04
cg26277237	9	631910	KANK1	-15.5495	0.00002	-0.0070	0.0019	2.17E-04
cg17944885	19	12114920	ZNF20;ZNF788P	-20.7212	0.00000	-0.0134	0.0031	1.26E-05

Breeze et al (African Americans only)						Discovery		
CpG associated with eGFR	Chr	Position (hg38)	Gene	Effect estimate	p value	Effect estimate	SE	p value
cg04682977	7	1023075	C7orf50;MIR339	-42.0636	0.0001518	-0.0091	0.0026	4.20E-04
cg26277237	9	631910	KANK1	-48.5529	3.967E-07	-0.0070	0.0019	2.17E-04
cg17944885	19	12114920	ZNF20;ZNF788	-50.5598	2.235E-06	-0.0134	0.0031	1.26E-05

Chen et al 2023							Discovery		
CpG	Chr	Position (bp)	Gene	Effect estimate	SE	P-value	Effect estimate	SE	p value
cg17944885	19	12225735	ZNF788/ZNF20	−1.30	0.24	3.01 × 10−8	-0.0134	0.0031	1.26E-05

Schlosser et al								Discovery		
CpG associated with eGFR	Chromosome	Position (b37)	Nearest Gene	Effect size (multi-ancestry meta-analysis)	P-value (multi-ancestry meta-analysis)	Effect size (AA-meta-analysis)	P-value (AA-meta-analysis)	Effect estimate	SE	p value
cg00501876	3	39193251	*CSRNP1*	-8.980e-05	1.332e-17	-0.00014	1.21104E-07	-0.0068	0.0019	3.79E-04
cg04540493	16	47048265	*DNAJA2*	-7.150e-05	1.553e-14	-9.1E-05	3.29274E-05	-0.0086	0.0024	3.45E-04
cg04578362	2	145592759	*TEX41*	-7.872e-05	2.701e-09	-9.3E-05	0.003175968	-0.0116	0.0021	2.25E-08
cg06158227	15	43662311	*ZSCAN29*	-1.082e-04	8.736e-20	-0.00013	1.74957E-09	-0.0143	0.0031	2.98E-06
cg11544657	1	9968130	*CTNNBIP1*	-8.207e-05	9.563e-13	-0.00012	8.19837E-06	-0.0073	0.0018	7.66E-05
cg14029001	6	41998500	*CCND3*	-8.023e-05	5.784e-12	-9.4E-05	0.000193467	-0.0091	0.0021	1.54E-05
cg17944885	19	12225735	*ZNF788*	-1.748e-04	8.742e-41	-0.00014	1.24479E-09	-0.0134	0.0031	1.26E-05
cg19946376	3	184035137	*EIF4G1*	-7.683e-05	1.689e-13	-9.1E-05	6.54598E-05	-0.0062	0.0016	1.37E-04
cg25570328	2	108903952	*SULT1C2*	-8.819e-05	7.889e-14	-5.7E-05	0.126925726	-0.0077	0.0019	4.82E-05

Supplementary Table 6: DNA methylation signals that was associated with gene expression, from the BIOS-BBRMI database ('cis-EQTMs independent top effects' dataset) ($p<0.05$)

	BIOS-BBRMI database										
Cpg	PValue	SNPChr	SNPChrPos	ProbeName	Cis Trans	SNP Type	Allele Assessed	Overall Z Score	HGNC Name	Beta (SE)	Fold Change
cg14849578	4.25E-07	12	1.25E+08	ENSG00000073060	cis	C/T	C	-5.0573316	SCARB1	-0.0955045 (0.0391344);-0.0605458 (0.0401197);-0.1406234 (0.0389837);-0.217072 (0.0723575)	0.000114

Supplementary Table 7A: Gene ontology biological processes at p<0.05

No	Term	Overlap	P-value	Adjusted P-value	Old P-value	Old Adjusted P-value	Odds Ratio	Combined Score	Genes
1	Organelle Organization (GO:0006996)	17/393	8.41E-06	0.008573	0	0	3.785475	44.23584	CUL7;YTHDF2;NUMA1;MAST4;SEC16A; LRRK2;NOL3;SIPA1L3;SYNE1;RAB43; NCKIPSD;BAG5;RANBP10;TRAPPC12; RAB38;KIFAP3;AJUBA
2	Regulation Of Transcription By RNA Polymerase II (GO:0006357)	46/1935	1.24E-05	0.008573	0	0	2.118148	23.92416	BARHL1;ZNF496;ZNF232;HDAC4;CSRNP1; CD81;GATA6;JADE1;CHD6;CCDC106; AEBP1;ZBTB4;ZMYM5;TTC21B;NAMPT; CAMTA1;APBB2;BRD8;NKX2-3;TGIF2; CDX2;ZBTB16;DNMT3A;IRX4;SOX13;EBF1; FOXN4;ZIC4;EVX2;TRERF1;TBX4;RNF40; RUNX1;FAM220A;NCOR2;CDK9;DMRTA2; ZEB1;WT1;NFIC;MAFG;IRF2;CDH13;LHX5; TCF4;SSBP3
3	Monoacylglycerol Metabolic Process (GO:0046462)	41365	1.46E-05	0.008573	0	0	36.21878	403.3703	FAAH;MOGAT2;ABHD6;MGLL
4	Monoacylglycerol Catabolic Process (GO:0052651)	45080	3.56E-05	0.015712	0	0	81.25926	832.3805	FAAH;ABHD6;MGLL
5	Cellular Response To Organic Substance (GO:0071310)	29768	6.26E-05	0.022126	0	0	7.761782	75.11896	GHR;CDK9;NPFFR1;SYK;GDF1;LDLRAP1; RAMP1
6	Protein-Containing Complex Assembly (GO:0065003)	13/307	0.00012	0.035207	0	0	3.666548	33.11342	HIP1;LRRK2;KLHL12;NOL3;PLEKHA7; TRAPPC12;GEMIN5;TCF4;TUBGCP4;KIFAP3; WASF1;ASB2;SHANK1
7	Regulation Of DNA-templated Transcription (GO:0006355)	41/1841	0.000161	0.040578	0	0	1.953712	17.06585	BARHL1;ZNF496;ZNF232;HDAC4;CSRNP1; OGG1;GATA6;JADE1;CREBL2;CCDC106; ZBTB4;TTC21B;CAMTA1;APBB2;BRD8; NKX2-3;TLE2;TGIF2;CDX2;ZBTB16;DNMT3A; IRX4;SOX13;EBF1;FOXN4;ZIC4;EVX2;TRERF1; TBX4;RUNX1;NCOR2;DMRTA2;ZEB1;CDK5; WT1;NFIC;MAFG;IRF2;LHX5;TCF4;AJUBA

Supplementary Table 7B: Gene ontology molecular processes at p<0.05

No	Term	Overlap	P-value	Adjusted P-value	Old P-value	Old Adjusted P-value	Odds Ratio	Combined Score	Genes
1	Signaling Receptor Complex Adaptor Activity (GO:0030159)	16193	0.000198	0.031501	0	0	10.46823	89.28778	LRRK2;TRADD;LDLRAP1;SHANK2;SHANK1
2	Cis-Regulatory Region Sequence-Specific DNA Binding (GO:0000987)	27/1056	0.000285	0.031501	0	0	2.213003	18.06749	ZNF496;ZNF232;HDAC4;GATA6;CCDC106;ZBTB4;NKX2-3; TGIF2;CDX2;ZBTB16;DNMT3A;IRX4;SOX13;EBF1;FOXN4; ZIC4;EVX2;TBX4;RUNX1;DMRTA2;ZEB1;WT1;NFIC;MAFG; ZNF30;IRF2;TCF4
3	RNA Polymerase II Transcription Regulatory Region Sequence-Specific DNA Binding (GO:0000977)	29/1178	0.000317	0.031501	0	0	2.132809	17.18189	BARHL1;ZNF496;ZNF232;HDAC4;GATA6;CCDC106;AEBP1; ZBTB4;NKX2-3;TGIF2;CDX2;ZBTB16;DNMT3A;IRX4;SOX13; EBF1;ZIC4;EVX2;TBX4;RUNX1;DMRTA2;ZEB1;WT1;NFIC;MAFG; ZNF30;IRF2;LHX5;TCF4
4	Microtubule Minus-End Binding (GO:0051011)	45263	0.000371	0.031501	0	0	27.08025	213.944	MACF1;NUMA1;TUBGCP4
5	mRNA Binding (GO:0003729)	11/270	0.000545	0.03412	0	0	3.50413	26.33281	NYNRIN;YTHDF2;ESRP2;DND1;GEMIN5;IGF2BP2;NOL3;MYH10; RNF40;ZNF385A;EDC3
6	Acylglycerol Lipase Activity (GO:0047372)	41699	0.000602	0.03412	0	0	22.15488	164.2797	FAAH;ABHD6;MGLL
7	RNA Polymerase II Cis-Regulatory Region Sequence-Specific DNA Binding (GO:0000978)	26/1080	0.000894	0.043327	0	0	2.071533	14.54089	ZNF496;ZNF232;HDAC4;GATA6;CCDC106;ZBTB4;NKX2-3; TGIF2;CDX2;ZBTB16;DNMT3A;IRX4;SOX13;EBF1;ZIC4;EVX2; TBX4;RUNX1;DMRTA2;ZEB1;WT1;NFIC;MAFG;ZNF30;IRF2;TCF4
8	Sequence-Specific DNA Binding (GO:0043565)	19/701	0.001158	0.043327	0	0	2.320005	15.68663	BARHL1;HDAC4;CSRNP1;TGIF2;CDX2;SOX13;GATA6;ZIC4;EVX2; ZBTB4;SMG6;DMRTA2;WT1;NFIC;MAFG;IRF2;LHX5;TCF4;NKX2-3
9	Transcription Coregulator Binding (GO:0001221)	35582	0.001262	0.043327	0	0	5.38975	35.97859	CDK9;NEK6;ZBTB16;GATA6;CHD6;TRERF1
10	Glutamate Receptor Binding (GO:0035254)	43160	0.001302	0.043327	0	0	16.24444	107.9306	HIP1;SHANK2;SHANK1
11	Phosphatidylinositol Binding (GO:0035091)	36312	0.001402	0.043327	0	0	5.27344	34.64667	SCARB1;HIP1;NUMA1;RCSD1;SNX31;PARD3B
12	aminoacyl-tRNA Ligase Activity (GO:0004812)	43525	0.001532	0.043398	0	0	15.22859	98.70222	LRRC47;CARS2;FARS2

Supplementary Table 8: Top 10 pathways from the Reactome Pathway Database

No	Term	Overlap	P-value	Adjusted P-value	Old P-value	Old Adjusted P-value	Odds Ratio	Combined Score
1	Signal Transduction R-HSA-162582	56/2395	2.01E-06	0.00136	0	0	2.119605	27.80189
2	Effects Of PIP2 Hydrolysis R-HSA-114508	46478	0.000312	0.105503	0	0	14.16503	114.341
3	Diseases Of Metabolism R-HSA-5668914	10/240	0.000813	0.164385	0	0	3.579893	25.47082
4	Immune System R-HSA-168256	39/1885	0.000973	0.164385	0	0	1.796141	12.45704
5	Disease R-HSA-1643685	35/1667	0.001391	0.164926	0	0	1.81403	11.9319
6	Arachidonate Production From DAG R-HSA-426048	45048	0.001464	0.164926	0	0	54.00615	352.4814
7	Interleukin-10 Signaling R-HSA-6783783	16163	0.00205	0.184039	0	0	8.139628	50.38332
8	RUNX1 Regulates Transcription Of Genes Involved In BCR Signaling R-HSA-8939245	45079	0.002178	0.184039	0	0	40.50308	248.2579
9	Defective Factor VIII Causes Hemophilia A R-HSA-9662001	45109	0.003024	0.211532	0	0	32.40123	187.96
10	Rab Regulation Of Trafficking R-HSA-9007101	6/118	0.003399	0.211532	0	0	4.375668	24.87268

Supplementary Table 9: DNA methylation signals that overlapped with traits from the GWAS 2023 database, $p < 0.05$

No	Term	Overlap	P-value	Adjusted P-value	Odds Ratio	Combined Score	Genes
1	Height	131/5412	1.7E-16	3.1E-13	2.7E+00	9.7E+01	NPFFR1;DGKD;CD81;GOLIM4;ADARB2;TSKU;ZFYVE28;FADS3;GOLGA3;IPO8;MPRIP;FAM3C;KIFAP3;BAHCC1;PRKCE;LOXL1-AS1;CLEC11A;FBXO17;SOX13;EBF1;UBE2E2;FRMD4A;VWA5B1;RUNX1;SPATA13;NR2F2-AS1;AMPH;LY86-AS1;CARS2;CARD11;MAD1L1;MACF1;FUT10;KLHL12;IQGAP2;ZBTB4;LPP;SCUBE2;PDGFD;SERPINH1;MOV10L1;CAMTA1;SLC15A4;MYH10;TRPM3;ABCA1;LINC01088;STARD13;SLC10A7;TNFSF12;NEK6;ZBTB16;SEC16A;GDF1;FOXN4;TRERF1;CDK9;PTK7;RANBP10;PEMT;TCF4;ZNF496;CSRNP1;HIP1;NUMA1;MAST4;SLC44A2;CCDC126;MEGF11;DOCK9;LRRK2;LRRK1;CHD6;NAT10;SLC2A2;AFF3;PPP1R9A;SIPA1L3;FARS2;SMG6;SYNE1;LFNG;RPTOR;GHR;ADGRE2;IMMP2L;GRM4;SH3PXD2A;SLC22A18;TNFAIP8L1;ZNF385A;JAK1;ABCC1;CAMK1D;DUSP1;CHPF2;DNMT3A;GAB1;NAV2;CKAP4;PARD3B;NCOR2;CDH13;L3MBTL4;MGLL;LINC01258;SHANK2;SDCCAG8;HDAC4;PAPLN;PRR5L;PAPSS2;NKD1;APBB2;LRRC8A;BRD8;IGF2BP2;WASF1;MUC4;CNNM4;YTHDF2;DNAJC5B;ERAP1;SYT16;ZIC4;PLEKHA7;NIN;PPP2R2C;NFIC;SSBP3;LGR4
2	Smoking Initiation	41/1049	6.5E-11	6.0E-08	3.6E+00	8.4E+01	HDAC4;MACF1;MRPS35;MAST4;MEGF11;SEZ6;CHD6;CCDC106;AFF3;SIPA1L3;LPP;SMG6;FBXO42;GOLGA3;IMMP2L;PPFIA1;CAMTA1;APBB2;TRPM3;RABGAP1L;SLC10A7;CAMK1D;TRPC4;PRKCE;ZBTB16;PLEKHA5;ZIC4;NAV2;MYO19;DUSP6;PLEKHA7;CKAP4;NCOR2;PCCA;PTK7;NR2F2-AS1;CDH13;TCF4;MGLL;MAD1L1;SHANK2
3	Educational Attainment	52/1786	5.5E-09	3.4E-06	2.7E+00	5.1E+01	HIP1;MAST4;MEGF11;JADE1;CHD6;AFF3;TSKU;SYNE1;RPTOR;GRM4;SH3PXD2A;TRAPPC12;BAHCC1;CAMK1D;PRKCE;LOXL1-AS1;DNMT3A;UBE2E2;VWA5B1;DUSP6;CKAP4;PARD3B;PCCA;NR2F2-AS1;CDH13;RAB38;LY86-AS1;LINC01258;MAD1L1;SHANK2;SDCCAG8;HDAC4;EXD3;FUT11;ABHD6;ZBTB4;LPP;MGAT5;ST8SIA5;CAMTA1;APBB2;CILP2;NCAM2;TRPM3;LINC01088;RABGAP1L;ZBTB16;PLEKHA5;TRAPPC9;PPP2R2C;NFIC;TCF4

4	Body Mass Index	48/1672	3.6E-08	1.6E-05	2.6E+00	4.5E+01	BLK;MACF1;ZCCHC7;EXD3;HIP1;MAST4;MEGF11;CHD6;SLC2A2;AFF3;ITIH1;LPP;FARS2;SMG6;FBXO42;RPTOR;GOLGA3;CAMTA1;TDH;IGF2BP2;TRPM3;RABGAP1L;SLC10A7;PRKCE;NEK6;ZBTB16;DNMT3A;ERAP1;EBF1;GAB1;PLEKHA5;SYT16;MYO19;DUSP6;PLEKHA7;PARD3B;NCOR2;NFIC;CACHD1;SPATS2L;CDH13;LHX5;TCF4;SSBP3;LGR4;MAD1L1;SHANK2;SDCCAG8
5	Diastolic Blood Pressure	28/716	8.2E-08	3.0E-05	3.5E+00	5.7E+01	HDAC4;EXD3;HIP1;MAST4;CHD6;LPP;SLC5A5;SMG6;SH3PXD2A;CAMTA1;BAHCC1;DUSP1;TRPC4;PRKCE;FBXO17;ERAP1;EBF1;UBE2E2;FRMD4A;TRAPPC9;SORBS3;PLEKHA7;NCOR2;NIN;PTK7;CDH13;CARS2;SDCCAG8
6	Migraine	11/110	1.2E-07	3.5E-05	9.2E+00	1.5E+02	NCOR2;MACF1;RABGAP1L;DCLRE1C;NR2F2-AS1;CAMTA1;FRMD4A;ADARB2;ZBTB4;RUNX1;SMG6
7	Serum Total Protein Level	17/299	2.0E-07	5.2E-05	5.1E+00	7.8E+01	BLK;MACF1;DGKD;NUMA1;CHPF2;SEC16A;LRRK1;EBF1;IQGAP2;AFF3;PPOX;RUNX1;SMG6;NYNRIN;SH3PXD2A;CARD11;HLA-DQB1
8	Adult Body Size	18/337	2.2E-07	5.2E-05	4.8E+00	7.3E+01	MACF1;RABGAP1L;SLC10A7;MAST4;MEGF11;LOXL1-AS1;PLEKHA5;SYT16;SLC2A2;AFF3;LPP;RPTOR;CAMTA1;CDH13;SSBP3;TRPM3;MAD1L1;SDCCAG8
9	Smoking Initiation (Ever Regular Vs Never Regular)	14/215	4.9E-07	9.9E-05	5.8E+00	8.5E+01	TRPC4;ZBTB16;SEZ6;ZIC4;NAV2;AFF3;SMG6;GOLGA3;IMMP2L;HEATR5A;PCCA;NR2F2-AS1;CAMTA1;MAD1L1
10	Self-reported Math Ability (MTAG)	18/388	1.7E-06	3.2E-04	4.1E+00	5.4E+01	EXD3;HIP1;GOLIM4;JADE1;AFF3;PPP1R9A;SIPA1L3;MYO19;RAPGEFL1;DUSP6;GOLGA3;NFIC;CDH13;APBB2;CILP2;TCF4;MAD1L1;SDCCAG8
11	Vertex-wise Cortical Surface Area	18/405	3.1E-06	4.9E-04	3.9E+00	4.9E+01	MACF1;DOCK9;ZBTB16;EBF1;ZIC4;NAV2;IQGAP2;PLEKHA7;SMG6;FBXO42;PARD3B;PCCA;PDGFD;CACHD1;TCF4;AJUBA;MYH10;SSBP3
12	Vertex-wise Sulcal Depth	21/533	3.2E-06	4.9E-04	3.5E+00	4.4E+01	MACF1;MAST4;DOCK9;ZBTB16;EBF1;ZIC4;NAV2;RCSD1;SIPA1L3;PLEKHA7;SMG6;ADAMTS13;PCCA;PDGFD;CACHD1;TRAPPC12;PPFIA1;CAMTA1;APBB2;TCF4;MYH10
13	Plateletcrit	20/493	3.6E-06	5.0E-04	3.6E+00	4.5E+01	CSRNP1;STARD13;DGKD;SYK;MAST4;PRKCE;TNFSF12;ZBTB16;LRRK2;ERAP1;IQGAP2;AFF3;RUNX1;SMG6;NFIC;SPATA13;KCNQ1DN;IGF2BP2;MAD1L1;JAK1
14	Post Bronchodilator FEV1	10/135	6.8E-06	8.8E-04	6.6E+00	7.9E+01	LFNG;FCER2;MAST4;EBF1;CAMTA1;AMPH;TRPM3;SHANK2;MGLL;SYNE1
15	Glaucoma (Primary Open-Angle)	30164	7.6E-06	8.8E-04	8.9E+00	1.0E+02	DGKD;APBB2;ITIH1;LPP;EXOC2;PLEKHA7;FARS2;SMG6

16	Brain Morphology (MOSTest)	18/432	7.6E-06	8.8E-04	3.6E+00	4.3E+01	MACF1;MAST4;DOCK9;ZBTB16;ZIC4;NAV2;AFF3;PLEKHA7;SMG6;SH3PXD2A;PCCA;PTK7;PDGFD;CAMTA1;TCF4;BAHCC1;MYH10;SSBP3
17	Glycated Hemoglobin Levels	14/275	8.7E-06	9.4E-04	4.5E+00	5.2E+01	MACF1;ABCC1;EBF1;SEZ6;UBE2E2;SLC2A2;AEBP1;FADS3;ADAMTS13;NFIC;CYP2R1;ATP11AUN;IGF2BP2;MAD1L1
18	Body Mass Index (MTAG)	24/718	1.0E-05	1.1E-03	2.9E+00	3.4E+01	MACF1;ZCCHC7;RABGAP1L;SLC10A7;HIP1;MAST4;MEGF11;NEK6;DNMT3A;EBF1;SYT16;SLC2A2;ITIH1;DUSP6;SMG6;PARD3B;RPTOR;CAMTA1;CDH13;SSBP3;TRPM3;LGR4;MAD1L1;SDCCAG8
19	Bipolar Disorder	11/180	1.5E-05	1.4E-03	5.4E+00	6.0E+01	RPTOR;ZCCHC7;CAMK1D;SLC44A2;SPATS2L;CDH13;ITIH1;MAD1L1;ANKHD1;SHANK2;SYNE1
20	Self-reported Math Ability	14/289	1.5E-05	1.4E-03	4.2E+00	4.7E+01	EXD3;GOLIM4;JADE1;PPP1R9A;AFF3;SIPA1L3;MYO19;GOLGA3;NFIC;CDH13;CILP2;TCF4;MAD1L1;SDCCAG8
21	Schizophrenia	26/841	1.7E-05	1.5E-03	2.7E+00	3.0E+01	ZCCHC7;ITIH1;SIPA1L3;LPP;SMG6;SYNE1;ZFYVE28;RPTOR;IMMP2L;LINC01088;RABGAP1L;TRPC4;SYT16;MYO19;PLEKHA7;NCOR2;NIN;HECW2;SPATS2L;CDH13;TCF4;PLCB2;MGLL;MAD1L1;HLA-DQB1;SDCCAG8
22	Drinks Per Week	16/384	2.4E-05	2.0E-03	3.6E+00	3.9E+01	LINC01088;RABGAP1L;MAST4;TNFSF12;JADE1;GDF1;UBE2E2;FRMD4A;ZIC4;AFF3;FBXO42;RPTOR;NCOR2;TCF4;MAD1L1;SDCCAG8
23	Platelet Distribution Width	16/387	2.7E-05	2.0E-03	3.6E+00	3.8E+01	MACF1;EXD3;SYK;SLC44A2;DOCK9;LRRK2;IQGAP2;LPP;NYNRIN;NCOR2;MOV10L1;CAMTA1;LDLRAP1;SSBP3;NKX2-3;SDCCAG8
24	Non-albumin Protein Levels	11/192	2.7E-05	2.0E-03	5.0E+00	5.3E+01	FADS3;BLK;NUMA1;CHPF2;TNFSF12;LRRK1;EBF1;AFF3;PPOX;EXOC2;CARD11
25	Highest Math Class Taken (MTAG)	20/568	2.8E-05	2.0E-03	3.1E+00	3.2E+01	EXD3;RABGAP1L;HIP1;CAMK1D;MAST4;JADE1;AFF3;PPP1R9A;ZBTB4;RAPGEFL1;DUSP6;PCCA;NFIC;CAMTA1;CDH13;TCF4;BRD8;NCAM2;MAD1L1;SDCCAG8
26	Adverse Response To Drug	19146	4.1E-05	2.9E-03	1.1E+01	1.1E+02	IRF2;SOX13;NAV2;ADARB2;KIFAP3;MAD1L1
27	Hemoglobin A1c Levels	13/278	4.4E-05	3.0E-03	4.1E+00	4.1E+01	MACF1;ABCC1;YTHDF2;DGKD;SEC16A;EBF1;UBE2E2;SLC2A2;SMG6;NFIC;TCF4;IGF2BP2;MAD1L1
28	Severe COVID-19 Infection	15/361	4.5E-05	3.0E-03	3.6E+00	3.6E+01	KSR1;MEGF11;UBE2E2;PRR5L;ADARB2;RCSD1;FARS2;ZFYVE28;IMMP2L;ADGRG1;NR2F2-AS1;GSG1L;CAMTA1;CDH13;NCAM2
29	Venous Thromboembolism	8/108	5.7E-05	3.6E-03	6.6E+00	6.4E+01	ADAMTS13;VWF;SLC44A2;GEMIN5;NAV2;NCAM2;KIFAP3;SMG6
30	Vertex-wise Cortical Thickness	15/372	6.3E-05	3.9E-03	3.5E+00	3.4E+01	MACF1;DOCK9;ZBTB16;EBF1;ZIC4;NAV2;SNX31;PLEKHA7;SMG6;SH3PXD2A;PCCA;CAMTA1;TCF4;FAM3C;SSBP3

31	Cortical Surface Area	17/464	6.8E-05	4.1E-03	3.2E+00	3.0E+01	MACF1;MAST4;DOCK9;ZBTB16;EBF1;ZIC4;NAV2;IQGAP2;RCSD1;PLEKHA7;SMG6;PARD3B;PCCA;PDGFD;CACHD1;TCF4;SSBP3
32	Metabolite Levels	24/815	7.8E-05	4.5E-03	2.6E+00	2.4E+01	ABCA1;HDAC4;MAST4;DNAJC5B;FUT10;GOLIM4;SYT16;FRMD4A;NAV2;ADARB2;AFF3;ANKHD1;CKAP4;RUNX1;FADS3;PPP2R2C;PDGFD;SPOCK2;SPATA13;SPATS2L;CDH13;IGF2BP2;TRPM3;SDCCAG8
33	Balding Type 1	9/149	9.8E-05	5.1E-03	5.3E+00	4.9E+01	NCOR2;NIN;LRRK1;EBF1;TCF4;AFF3;LGR4;RUNX1;SMG6
34	SAPHO Syndrome	45141	9.8E-05	5.1E-03	4.9E+01	4.5E+02	PAPLN;FBXO17;APBA2
35	Sphingomyelin (D18:1/25:0, D19:0/24:1, D20:1/23:0, D19:1/24:0) Levels In Elite Athletes	45141	9.8E-05	5.1E-03	4.9E+01	4.5E+02	NR2F2-AS1;TRAPPC9;FARS2
36	Highest Math Class Taken	10/185	1.0E-04	5.1E-03	4.7E+00	4.3E+01	EXD3;HIP1;ESRP2;CDH13;TCF4;AFF3;PPOX;DUSP6;MAD1L1;SDCCAG8
37	Educational Attainment (MTAG)	21/675	1.0E-04	5.1E-03	2.7E+00	2.5E+01	EXD3;HIP1;CAMK1D;MAST4;ZBTB16;AFF3;ZBTB4;RAPGEFL1;TSKU;RPTOR;SH3PXD2A;PCCA;CAMTA1;CDH13;RAB38;TCF4;BRD8;NCAM2;TRPM3;MAD1L1;SDCCAG8
38	Morningness	9/151	1.1E-04	5.2E-03	5.2E+00	4.8E+01	HDAC4;ZCCHC7;EXD3;RABGAP1L;SLC44A2;HECW2;ZBTB16;UBE2E2;TCF4
39	Smoking Initiation (Ever Regular Vs Never Regular) (MTAG)	14/348	1.1E-04	5.3E-03	3.5E+00	3.2E+01	MEGF11;TRPC4;ZBTB16;SEZ6;ZIC4;NAV2;AFF3;SMG6;GOLGA3;IMMP2L;NR2F2-AS1;CAMTA1;MGLL;MAD1L1
40	Chronic Obstructive Pulmonary Disease Liability (Machine Learning-Based Score)	11/226	1.2E-04	5.5E-03	4.2E+00	3.8E+01	SCUBE2;SLC10A7;SH3PXD2A;TNFSF12;DNMT3A;RANBP10;SPATS2L;FRMD4A;FAM3C;AFF3;SMG6
41	Coronary Artery Disease	16/441	1.2E-04	5.6E-03	3.1E+00	2.8E+01	ABCA1;SCARB1;MAST4;PRKCE;PPP1R9A;PLEKHA7;SMG6;ZEB1;PTK7;WT1;SERPINH1;CDH13;IGF2BP2;PLCB2;TRPM3;MAD1L1
42	Waist Circumference Adjusted For Body Mass Index	24/842	1.3E-04	5.6E-03	2.5E+00	2.2E+01	ABCA1;BLK;MACF1;ABCC1;TNFSF12;LOXL1-AS1;DNMT3A;EBF1;PRR5L;ADARB2;ITIH1;ZBTB4;TBX4;SMG6;NCOR2;NCKIPSD;GRM4;SH3PXD2A;SERPINH1;CAMTA1;IGF2BP2;NES;TRPM3;ZNF385A
43	Venous Thromboembolism Adjusted For Sickle Cell Variant rs77121243-T	15097	1.4E-04	6.0E-03	1.1E+01	1.0E+02	ABCC1;PRKCE;IQGAP2;TRPM3;PARD3B
44	Prostate Cancer (Advanced)	45172	1.5E-04	6.1E-03	4.1E+01	3.6E+02	ADGRG1;TCF4;MAD1L1

45	Mean Spheric Corpuscular Volume	17/496	1.5E-04	6.2E-03	3.0E+00	2.6E+01	SCARB1;ABCC1;SYK;SLC44A2;ZBTB16;EBF1;IQGAP2;AFF3;LPP;RUNX1;LIMD2;NCOR2;WT1;SPATA13;PEMT;IGF2BP2;NKX2-3
46	Platelet Count	24/859	1.7E-04	6.9E-03	2.4E+00	2.1E+01	CSRNP1;DGKD;SYK;MAST4;VWF;SLC44A2;PRKCE;ZBTB16;LRRK2;ERAP1;EBF1;IQGAP2;MAPK12;RUNX1;SMG6;NFIC;CAMTA1;IGF2BP2;LDLRAP1;SSBP3;MGLL;MAD1L1;JAK1;HLA-DQB1
47	Heel Bone Mineral Density	25/914	1.8E-04	6.9E-03	2.4E+00	2.0E+01	BLK;HDAC4;MACF1;IFITM1;DOCK9;ZBTB4;PAPSS2;SMG6;SYNE1;GHR;FAM3C;IGF2BP2;BAHCC1;PRKCE;NEK6;ZBTB16;DNMT3A;EBF1;TBX4;RUNX1;HECW2;NFIC;SPATA13;TCF4;LGR4
48	Sex Hormone-Binding Globulin Levels Adjusted For BMI	17/503	1.8E-04	6.9E-03	2.9E+00	2.5E+01	MACF1;CSRNP1;DGKD;ZBTB16;DNMT3A;GAB1;PRR5L;ITIH1;NYNRIN;GHR;NFIC;NR2F2-AS1;IRF2;IGF2BP2;CARS2;LINC01258;MAD1L1
49	Male-pattern Baldness	12/279	1.9E-04	6.9E-03	3.7E+00	3.2E+01	NCOR2;HDAC4;NIN;NFIC;LRRK1;EBF1;TCF4;AFF3;EXOC2;LGR4;RUNX1;SMG6
50	Mean Platelet Volume	18/554	1.9E-04	6.9E-03	2.8E+00	2.4E+01	NUMA1;MAST4;VWF;ZBTB16;LRRK2;ERAP1;EBF1;IQGAP2;AFF3;RUNX1;EDC3;LFNG;NIN;MYO1C;CAMTA1;LRRC8A;LDLRAP1;SDCCAG8
51	Eosinophil Counts	21/706	1.9E-04	6.9E-03	2.6E+00	2.2E+01	FAAH;NEK6;SEC16A;FBXO17;TRERF1;SIPA1L3;LPP;RAPGEFL1;NKD1;RASGRP4;RUNX1;NCOR2;NCKIPSD;MGAT5;NFIC;ASB2;BAHCC1;EXOC2;MGLL;JAK1;HLA-DQB1
52	Cortical Thickness	14/367	2.0E-04	7.0E-03	3.3E+00	2.8E+01	MACF1;MAST4;DOCK9;ZBTB16;EBF1;ZIC4;NAV2;SNX31;PLEKHA7;SMG6;PCCA;CAMTA1;TCF4;LGR4
53	Systemic Lupus Erythematosus	12/281	2.0E-04	7.0E-03	3.7E+00	3.1E+01	BLK;LINC01088;RABGAP1L;HIP1;CAMK1D;KSR1;MGAT5;LRRK1;CREBL2;LPP;SLC15A4;HLA-DQB1
54	Basophil Count	10/202	2.1E-04	7.1E-03	4.3E+00	3.6E+01	ABCC1;HIP1;ZBTB16;CILP2;SLC15A4;RAPGEFL1;RASGRP4;CKAP4;RUNX1;HLA-DQB1
55	Age Of Smoking Initiation (MTAG)	36342	2.2E-04	7.4E-03	6.2E+00	5.2E+01	RABGAP1L;IMMP2L;PCCA;ZBTB16;NR2F2-AS1;SEZ6;MAD1L1
56	Neutrophil Percentage Of Granulocytes	7/100	2.4E-04	7.7E-03	6.2E+00	5.2E+01	ASB2;LPP;TRERF1;RASGRP4;EXOC2;RUNX1;JAK1
57	Atrial Fibrillation	9/168	2.4E-04	7.7E-03	4.7E+00	3.9E+01	NCOR2;MPRIP;SH3PXD2A;SYK;PCCA;TNFSF12;SPATS2L;NAV2;BEST3
58	Monocyte Percentage Of White Cells	13/330	2.4E-04	7.7E-03	3.4E+00	2.8E+01	SCARB1;ATP6V1G2;KSR1;ZBTB16;SEC16A;EBF1;TRERF1;LPP;RUNX1;LRRC31;BAHCC1;JAK1;CCR2
59	Reaction Time	11/247	2.6E-04	8.0E-03	3.8E+00	3.2E+01	RABGAP1L;IMMP2L;PRKCE;CHD6;SPATS2L;UBE2E2;CAMTA1;CDH13;NAV2;IQGAP2;PARD3B

60	Gut Microbiota (Bacterial Taxa)	46113	2.7E-04	8.0E-03	1.5E+01	1.2E+02	PLEKHA5;RAB38;SIPA1L3;EXOC2
61	Eczema	8/135	2.7E-04	8.0E-03	5.2E+00	4.3E+01	FLG;DNMT3A;UBE2E2;PRR5L;LPP;RUNX1;SMG6;JAK1
62	Body Size At Age 10	9/171	2.8E-04	8.0E-03	4.6E+00	3.7E+01	RPTOR;HDAC4;IMMP2L;NPFFR1;MAST4;EBF1;CAMTA1;SSBP3;SMG6
63	Monocyte Count	18/572	2.8E-04	8.0E-03	2.7E+00	2.2E+01	SCARB1;SYK;CDX2;KSR1;PRKCE;NEK6;ZBTB16;EBF1;LPP;RASGRP4;RUNX1;SMG6;NCOR2;IPO8;NFIC;TCF4;SSBP3;CCR2
64	Hip Circumference Adjusted For BMI	22/780	2.8E-04	8.0E-03	2.4E+00	2.0E+01	ABCA1;SCARB1;ABCC1;SLC44A2;DNMT3A;TRAPPC9;AFF3;TBX4;SMG6;SYNE1;PARD3B;NCOR2;GHR;GRM4;HECW2;NFIC;SERPINH1;PEMT;IGF2BP2;ERGIC3;BAHCC1;HLA-DQB1
65	BMI (Standard GWA)	8/136	2.8E-04	8.0E-03	5.1E+00	4.2E+01	FAM220A;RPTOR;MACF1;AFF3;DUSP6;LGR4;SMG6;SDCCAG8
66	Hemoglobin Concentration	16/475	2.9E-04	8.0E-03	2.9E+00	2.4E+01	LRRC47;EXD3;STARD13;ATP6V1G2;DGKD;PRKCE;UBE2E2;FRMD4A;AFF3;RUNX1;SMG6;NYNRIN;ADAMTS13;MPRIP;RANBP10;TUBGCP4
67	Externalizing Behaviour (Multivariate Analysis)	12/297	3.3E-04	9.1E-03	3.5E+00	2.8E+01	GOLGA3;IMMP2L;ZBTB16;NR2F2-AS1;PLEKHA5;CAMTA1;CDH13;TCF4;ZIC4;AFF3;MAD1L1;SMG6
68	Schizophrenia Vs ADHD (Ordinary Least Squares (OLS))	46844	3.6E-04	9.7E-03	1.4E+01	1.1E+02	IMMP2L;TCF4;MAD1L1;SDCCAG8
69	Triglyceride Levels	20/688	3.6E-04	9.7E-03	2.5E+00	2.0E+01	ABCA1;BLK;MACF1;SCARB1;TNFSF12;SEC16A;UBE2E2;SLC2A2;PRR5L;AFF3;ITIH1;LPP;ANKHD1;PARD3B;FADS3;PEMT;ERGIC3;L3MBTL4;SSBP3;HLA-DQB1
70	Depression Severity X Playing Computer Games Interaction	45263	3.7E-04	9.7E-03	2.7E+01	2.1E+02	CDH13;FRMD4A;L3MBTL4
71	Educational Attainment (Years Of Education)	20/693	4.0E-04	1.0E-02	2.5E+00	1.9E+01	HDAC4;EXD3;HIP1;CAMK1D;MAST4;ZBTB16;AFF3;RAPGEFL1;TSKU;SH3PXD2A;PCCA;FAM3B;CAMTA1;CDH13;CILP2;RAB38;TCF4;NCAM2;MAD1L1;SDCCAG8
72	Appendicular Lean Mass	21/755	4.6E-04	1.2E-02	2.4E+00	1.8E+01	MACF1;DGKD;SLC44A2;DOCK9;PRKCE;SEC16A;DNMT3A;CHD6;ITIH1;ZBTB4;RUNX1;SYNE1;NCOR2;GHR;MYO1C;NFIC;CAMTA1;TDH;BAHCC1;JAK1;SDCCAG8
73	Obesity (Early Onset Extreme)	41334	4.8E-04	1.2E-02	2.4E+01	1.9E+02	TCF4;FARS2;SDCCAG8
74	Whole Brain Restricted Isotropic Diffusion (Multivariate Analysis)	9/186	5.1E-04	1.3E-02	4.2E+00	3.2E+01	PCCA;DOCK9;ZBTB16;CHD6;ZIC4;NAV2;IQGAP2;SMG6;JAK1

75	Type 2 Diabetes	22/816	5.2E-04	1.3E-02	2.3E+00	1.8E+01	ABCA1;MACF1;DGKD;CAMK1D;SYK;MRPS35;DNMT3A;UBE2E2;SLC2A2;FRMD4A;AFF3;JAKMIP1;MYO19;TSKU;NYNRIN;RPTOR;PPP2R2C;SPOCK2;TCF4;IGF2BP2;MAD1L1;HLA-DQB1
76	Whole Brain Free Water Diffusion (Multivariate Analysis)	7/114	5.3E-04	1.3E-02	5.4E+00	4.0E+01	SH3PXD2A;PCCA;CHD6;ZIC4;NAV2;IQGAP2;SMG6
77	Blood Protein Levels	22/817	5.3E-04	1.3E-02	2.3E+00	1.7E+01	ATP6V1G2;HIP1;VWF;KSR1;CCDC126;IL1R2;TNFSF12;CLEC11A;ERAP1;ITIH1;PLEKHA7;CHIT1;GHR;FCER2;ADAMTS13;ESRP2;PDGFD;SPOCK2;FAM3B;MAN1C1;CDH13;NCAM2
78	HDL Cholesterol	12/314	5.5E-04	1.3E-02	3.3E+00	2.5E+01	ABCA1;MACF1;SCARB1;PDGFD;SLC22A18;NFIC;RANBP10;PEMT;TUBGCP4;AFF3;RUNX1;SMG6
79	Peripheral Arterial Disease (Traffic-Related Air Pollution Interaction)	30468	5.6E-04	1.3E-02	6.4E+00	4.8E+01	ABCA1;MACF1;PPP2R2C;SPATA13;FRMD4A;NAV2
80	Anorexia Nervosa, Attention-Deficit/Hyperactivity Disorder, Autism Spectrum Disorder, Bipolar Disorder, Major Depression, Obsessive-Compulsive Disorder, Schizophrenia, Or Tourette Syndrome (Pleiotropy)	30834	5.9E-04	1.4E-02	6.3E+00	4.7E+01	RPTOR;ZCCHC7;LINC01088;IMMP2L;TCF4;MAD1L1
81	Interleukin-2 Levels	41699	6.0E-04	1.4E-02	2.2E+01	1.6E+02	MEGF11;EBF1;ADARB2
82	Endometriosis Or Asthma (Pleiotropy)	11780	6.1E-04	1.4E-02	1.2E+01	8.6E+01	RABGAP1L;LPP;FARS2;RUNX1
83	Waist-hip Ratio	17/560	6.1E-04	1.4E-02	2.6E+00	1.9E+01	ABCA1;MAST4;PRR5L;AFF3;SNX31;RUNX1;SMG6;SCUBE2;IPO8;MPRIP;PTK7;WT1;PEMT;CDH13;IGF2BP2;LGR4;SDCCAG8
84	Morning Person	8/153	6.2E-04	1.4E-02	4.5E+00	3.3E+01	HDAC4;ZCCHC7;EXD3;RABGAP1L;ZBTB16;NEK6;UBE2E2;CAMTA1
85	Mean Arterial Pressure	10/232	6.3E-04	1.4E-02	3.7E+00	2.7E+01	RPTOR;HDAC4;ZCCHC7;PRKCE;EBF1;SEZ6;UBE2E2;BAHCC1;PLEKHA7;SMG6
86	Alcohol Consumption (Drinks Per Month) (UKB Data Field 1578, 4424)	12145	6.9E-04	1.5E-02	1.1E+01	8.2E+01	TNFSF12;CAMTA1;TCF4;BAHCC1

87	Waist-to-hip Ratio Adjusted For BMI	21/784	7.5E-04	1.5E-02	2.3E+00	1.7E+01	ABCA1;BLK;SCARB1;MAST4;DNMT3A;EBF1;PRR5L;AFF3;ITIH1;ZBTB4;RUNX1;SMG6;RAB43;IPO8;GRM4;MPRIP;PTK7;HECW2;PEMT;IGF2BP2;HLA-DQB1
88	C-reactive Protein Levels	14/420	7.5E-04	1.5E-02	2.9E+00	2.1E+01	ABCA1;MACF1;SCARB1;DUSP1;NEK6;EBF1;UBE2E2;SLC2A2;TRERF1;NYNRIN;CACHD1;CAMTA1;TCF4;JAK1
89	Schizophrenia (MTAG)	32295	7.6E-04	1.5E-02	6.0E+00	4.3E+01	IMMP2L;SPATS2L;TCF4;ITIH1;MAD1L1;SDCCAG8
90	Resistance To COVID-19 Infection (Exposed Negative Vs Positive)	12/326	7.6E-04	1.5E-02	3.2E+00	2.3E+01	LINC01088;EXD3;MYBPC2;VWF;MEGF11;PRKCE;NR2F2-AS1;CDH13;FRMD4A;NCAM2;MYH10;L3MBTL4
91	Hypothyroidism	7/122	7.9E-04	1.6E-02	5.0E+00	3.6E+01	NEK6;SPATA13;CDH13;LRRC8A;LPP;JAK1;HLA-DQB1
92	Calcium Levels	11/283	8.0E-04	1.6E-02	3.3E+00	2.4E+01	NYNRIN;HDAC4;MACF1;DGKD;CAMK1D;NUMA1;SLC22A18;NR2F2-AS1;DNMT3A;RAB38;CNNM4
93	Lung Function (FEV1/FVC)	16/525	8.5E-04	1.6E-02	2.6E+00	1.8E+01	HDAC4;DGKD;CAMK1D;MRPS35;DOCK9;FRMD4A;NAV2;AFF3;SMG6;SH3PXD2A;NFIC;SPATA13;SPATS2L;SFTPA1;EXOC2;HLA-DQB1
94	Risk-taking Tendency (4-Domain Principal Component Model)	22037	8.5E-04	1.6E-02	7.4E+00	5.2E+01	PCCA;ZIC4;TCF4;BAHCC1;MAD1L1
95	Thyroid Stimulating Hormone Levels	22037	8.5E-04	1.6E-02	7.4E+00	5.2E+01	DNAJC5B;NEK6;SPATA13;IGF2BP2;LPP
96	High Density Lipoprotein Cholesterol Levels	19/683	8.6E-04	1.6E-02	2.4E+00	1.7E+01	ABCA1;MACF1;SCARB1;RABGAP1L;ERAP1;PRR5L;CCDC106;IQGAP2;AFF3;ITIH1;SMG6;FADS3;GRM4;PDGFD;SLC22A18;NFIC;NR2F2-AS1;RANBP10;PEMT
97	Plasma Copper Levels	45018	8.9E-04	1.7E-02	8.1E+01	5.7E+02	CAMK1D;IRF2
98	Brain-derived Neurotrophic Factor Levels	45018	8.9E-04	1.7E-02	8.1E+01	5.7E+02	CSRNP1;RUNX1
99	Depression	8/163	9.4E-04	1.8E-02	4.2E+00	2.9E+01	BLK;ZCCHC7;LINC01088;GRHPR;CDH13;TCF4;MAD1L1;SHANK2
100	Free Cholesterol To Total Lipids Ratio In Large VLDL	13241	9.6E-04	1.8E-02	1.0E+01	7.1E+01	ABCA1;SCARB1;SEC16A;HLA-DQB1
101	Bipolar I Disorder	23132	1.1E-03	1.9E-02	7.0E+00	4.8E+01	MYO19;MAD1L1;SHANK2;ANKHD1;SYNE1
102	Blood Pressure (Smoking Interaction)	42795	1.1E-03	2.0E-02	1.7E+01	1.2E+02	TRAPPC9;ZSWIM7;ADARB2
103	Vaginal Microbiome MetaCyc Pathway (ENTBACSYN-PWY|enterobactin Biosynthesis)	42795	1.1E-03	2.0E-02	1.7E+01	1.2E+02	MACF1;MEGF11;GEMIN5

104	Whole Brain Restricted Directional Diffusion (Multivariate Analysis)	8/167	1.1E-03	2.0E-02	4.1E+00	2.8E+01	MACF1;SH3PXD2A;PCCA;EBF1;ZIC4;NAV2;PLEKHA7;SMG6
105	Sum Eosinophil Basophil Counts	7/130	1.1E-03	2.0E-02	4.7E+00	3.2E+01	ASB2;LPP;TRERF1;RASGRP4;EXOC2;RUNX1;JAK1
106	Autism Spectrum Disorder, Attention Deficit-Hyperactivity Disorder, Bipolar Disorder, Major Depressive Disorder, And Schizophrenia (Combined)	14336	1.3E-03	2.3E-02	9.3E+00	6.2E+01	HDAC4;TCF4;MAD1L1;SYNE1
107	Triiodothyronine Levels	45048	1.5E-03	2.4E-02	5.4E+01	3.5E+02	PRKCE;RAB38
108	Acute Anterior Uveitis In Ankylosing Spondylitis	45048	1.5E-03	2.4E-02	5.4E+01	3.5E+02	ERAP1;KIFAP3
109	Corneal Endothelial Cell Shape (Percentage Of Hexagonally Shaped Cells)	45048	1.5E-03	2.4E-02	5.4E+01	3.5E+02	TCF4;APBA2
110	Pallidum Volume Change Rate	45048	1.5E-03	2.4E-02	5.4E+01	3.5E+02	CDH13;TRAPPC9
111	Subjective Response To Placebo Treatment In Childhood Asthma (Change In Cough/Wheeze)	45048	1.5E-03	2.4E-02	5.4E+01	3.5E+02	NAV2;FARS2
112	A Body Shape Index	12/353	1.5E-03	2.5E-02	2.9E+00	1.9E+01	ABCA1;BLK;NCKIPSD;GRM4;DNMT3A;PEMT;PRR5L;IGF2BP2;IQGAP2;AFF3;ITIH1;HLA-DQB1
113	Protein Quantitative Trait Loci (Liver)	22/887	1.5E-03	2.5E-02	2.1E+00	1.4E+01	MACF1;LINC01088;VWF;TRPC4;PRKCE;ZBTB16;ERAP1;GAB1;TTF2;TRAPPC9;NAV2;LPP;RUNX1;PARD3B;ZFYVE28;ADGRE2;GRM4;MGAT5;CDH13;AMPH;RAB38;CARD11
114	Knee Osteoarthritis	43525	1.5E-03	2.5E-02	1.5E+01	9.9E+01	FRMD4A;PARD3B;SMG6
115	Total Bilirubin Levels	8/177	1.6E-03	2.6E-02	3.9E+00	2.5E+01	SCARB1;DGKD;PRKCE;SLC22A18;EBF1;SIPA1L3;L3MBTL4;SMG6
116	Asthma	10/265	1.7E-03	2.7E-02	3.2E+00	2.1E+01	FLG;NEK6;FUT11;GAB1;UBE2E2;PRR5L;LPP;CARD11;RUNX1;HLA-DQB1
117	Hair Curvature (Quantitative)	43891	1.8E-03	2.8E-02	1.4E+01	9.1E+01	RPTOR;FRMD4A;ADARB2
118	Basophil Percentage Of White Cells	6/104	1.8E-03	2.8E-02	5.0E+00	3.2E+01	ABCC1;HIP1;SLC15A4;RASGRP4;CKAP4;RUNX1
119	Eosinophil Percentage Of Granulocytes	6/105	1.9E-03	2.9E-02	5.0E+00	3.1E+01	ASB2;LPP;TRERF1;EXOC2;RUNX1;JAK1

120	Systolic Blood Pressure	23/964	2.0E-03	3.0E-02	2.0E+00	1.3E+01	HDAC4;HIP1;MAST4;PRKCE;TNFSF12;ERAP1;EBF1;CHD6;MAT1A;TRAPPC9;CLN8;PLEKHA7;SMG6;RPTOR;NIN;SH3PXD2A;HEATR5A;WT1;NR2F2-AS1;CDH13;TCF4;BAHCC1;MAD1L1
121	DNA Methylation Variation (Age Effect)	8/184	2.0E-03	3.1E-02	3.7E+00	2.3E+01	ABCA1;RABGAP1L;HIP1;SH3PXD2A;PRKCE;CDH13;RUNX1;PARD3B
122	Respiratory Diseases	26785	2.0E-03	3.1E-02	6.0E+00	3.7E+01	FLG;PRR5L;LPP;CARD11;RUNX1
123	Caffeine Levels In Elite Athletes	45079	2.2E-03	3.2E-02	4.1E+01	2.5E+02	MEGF11;LRRK2
124	Rapid Automised Naming Of Digits	45079	2.2E-03	3.2E-02	4.1E+01	2.5E+02	CDH13;FRMD4A
125	Thalamus Volume Change Rate X Age Interaction (2Df)	45079	2.2E-03	3.2E-02	4.1E+01	2.5E+02	ABCC1;ADARB2
126	Response To Cyclophosphamide In Systemic Lupus Erythematosus With Lupus Nephritis	45079	2.2E-03	3.2E-02	4.1E+01	2.5E+02	SCARB1;CAMK1D
127	Tourette's Syndrome Or Obsessive-Compulsive Disorder	45079	2.2E-03	3.2E-02	4.1E+01	2.5E+02	TRPC4;CAMTA1
128	HDL Cholesterol Levels	14/473	2.3E-03	3.3E-02	2.5E+00	1.5E+01	ABCA1;MACF1;SCARB1;EBF1;AFF3;RAPGEFL1;SMG6;ADGRG1;PDGFD;SLC22A18;NFIC;NT5C1A;PEMT;HLA-DQB1
129	Chronic Kidney Disease	27515	2.3E-03	3.3E-02	5.8E+00	3.5E+01	SIPA1L3;PLCB2;LPP;MYO19;SDCCAG8
130	Lung Function (FVC)	13/425	2.5E-03	3.5E-02	2.6E+00	1.6E+01	ZBTB16;EBF1;GAB1;AFF3;VWA5B1;ZBTB4;SMG6;SYNE1;NCOR2;MYO1C;IGF2BP2;LGR4;MAD1L1
131	Chronotype	9/234	2.5E-03	3.5E-02	3.3E+00	2.0E+01	HDAC4;LINC01088;EXD3;RABGAP1L;SLC10A7;ZBTB16;NEK6;SYT16;TCF4
132	Medication Use For T2D (Number Of Purchases)	17258	2.6E-03	3.6E-02	7.6E+00	4.5E+01	MACF1;UBE2E2;IGF2BP2;MAD1L1
133	Adolescent Idiopathic Scoliosis	16/588	2.7E-03	3.7E-02	2.3E+00	1.4E+01	STARD13;PRKCE;FRMD4A;ADARB2;AFF3;BEST3;PARD3B;PPP2R2C;NR2F2-AS1;IRF2;ST8SIA5;SPATS2L;CDH13;LY86-AS1;RAMP1;L3MBTL4
134	Hypertension	7/151	2.7E-03	3.7E-02	4.0E+00	2.4E+01	RPTOR;NCOR2;NR2F2-AS1;ERAP1;EBF1;PLCB2;L3MBTL4
135	Disruptive Behavior (Multivariate Analysis)	44986	2.7E-03	3.7E-02	1.2E+01	7.2E+01	RPTOR;TCF4;MAD1L1
136	Cognitive Performance (MTAG)	11/331	2.8E-03	3.7E-02	2.8E+00	1.7E+01	EXD3;RABGAP1L;MAST4;CDH13;TCF4;NCAM2;AFF3;TSKU;DUSP6;MAD1L1;SDCCAG8

137	Phenylephrine Infusion Rate During Anesthesia	45109	3.0E-03	3.9E-02	3.2E+01	1.9E+02	FAM3C;IQGAP2
138	Serum Zinc Levels	45109	3.0E-03	3.9E-02	3.2E+01	1.9E+02	CAMK1D;SIPA1L3
139	Diffuse Plaques (SNP X SNP Interaction)	45109	3.0E-03	3.9E-02	3.2E+01	1.9E+02	CUL7;ADARB2
140	Phosphatidylcholine 40:6 [M+OAc]1- /Phosphatidylserine 44:5 [M-H]1- Levels	45109	3.0E-03	3.9E-02	3.2E+01	1.9E+02	FADS3;PEMT
141	Pregnanediol-3-glucuronide Levels In Elite Athletes	45109	3.0E-03	3.9E-02	3.2E+01	1.9E+02	CCDC78;ADARB2
142	Sardines Liking	45109	3.0E-03	3.9E-02	3.2E+01	1.9E+02	CDH13;TCF4
143	Coffee Type: Decaffeinated Coffee (Any Type) (UKB Data Field 1508 1)	45109	3.0E-03	3.9E-02	3.2E+01	1.9E+02	NCOR2;FAAH
144	Phosphatidylcholine Levels	17989	3.1E-03	3.9E-02	7.2E+00	4.2E+01	FADS3;BLK;ABCA1;SCARB1
145	Hodgkin's Lymphoma	17989	3.1E-03	3.9E-02	7.2E+00	4.2E+01	MEGF11;ERAP1;LPP;SHANK2
146	Major Depressive Disorder	8/197	3.1E-03	3.9E-02	3.5E+00	2.0E+01	LINC01088;ZCCHC7;ADGRE2;EXD3;TCF4;TRAPPC9;TRPM3;MAD1L1
147	Apolipoprotein A1 Levels	12/390	3.4E-03	4.2E-02	2.6E+00	1.5E+01	ABCA1;FADS3;MACF1;SCARB1;PDGFD;TNFSF12;SLC22A18;NFIC;PEMT;AFF3;ITIH1;SMG6
148	Photic Sneeze Reflex	45717	3.4E-03	4.3E-02	1.1E+01	6.3E+01	ADAMTS13;CAMTA1;PRR5L
149	R-warfarin Levels	30437	3.6E-03	4.4E-02	5.2E+00	2.9E+01	CAMTA1;IQGAP2;VWA5B1;L3MBTL4;FARS2
150	Pulse Pressure	17/664	3.7E-03	4.5E-02	2.2E+00	1.2E+01	HIP1;PRKCE;FUT10;TNFSF12;NEK6;EBF1;TRAPPC9;ITIH1;LPP;SMG6;RPTOR;NIN;MYO1C;HEATR5A;CDH13;TCF4;SDCCAG8
151	Number Of Sexual Partners	19085	3.8E-03	4.5E-02	6.8E+00	3.8E+01	BLK;ZIC4;TCF4;MAD1L1
152	Hematocrit	14/501	3.8E-03	4.5E-02	2.4E+00	1.3E+01	STARD13;RABGAP1L;DGKD;PRKCE;EBF1;UBE2E2;FRMD4A;AFF3;RUNX1;NCOR2;MPRIP;WT1;RANBP10;BAHCC1
153	Venous Thromboembolism Or Factor VII Levels (Pleiotropy)	46082	3.9E-03	4.5E-02	1.1E+01	5.9E+01	VWF;SLC44A2;TSKU
154	Microalbuminuria	46082	3.9E-03	4.5E-02	1.1E+01	5.9E+01	NUMA1;SPATA13;CAMTA1
155	Stem Cell Factor Levels	46082	3.9E-03	4.5E-02	1.1E+01	5.9E+01	ABCA1;PPP2R2C;FUT10
156	Immune Response To Smallpox Vaccine (IL-6)	46082	3.9E-03	4.5E-02	1.1E+01	5.9E+01	BLK;GOLGA3;RUNX1

157	Hemoglobin	13/449	3.9E-03	4.5E-02	2.5E+00	1.4E+01	STARD13;DGKD;PRKCE;UBE2E2;FRMD4A;AFF3;ZBTB4;RUNX1;SMG6;NYNRIN;NCOR2;MPRIP;RANBP10
158	Menarche (Age At Onset)	9/250	3.9E-03	4.5E-02	3.1E+00	1.7E+01	RPTOR;HIP1;PTK7;TCF4;ADARB2;AFF3;SSBP3;TRPM3;MAD1L1
159	Longevity	31168	4.0E-03	4.5E-02	5.1E+00	2.8E+01	ZFYVE28;RABGAP1L;NCAM2;MAD1L1;TRPM3
160	Oral Cavity Cancer	45140	4.0E-03	4.5E-02	2.7E+01	1.5E+02	NR2F2-AS1;HLA-DQB1
161	Bullous Pemphigoid	45140	4.0E-03	4.5E-02	2.7E+01	1.5E+02	MAST4;HLA-DQB1
162	Posterior Urethral Valves	45140	4.0E-03	4.5E-02	2.7E+01	1.5E+02	PTK7;SMG6
163	Autoimmune Traits	19815	4.3E-03	4.9E-02	6.5E+00	3.5E+01	SPATA13;LPP;JAK1;HLA-DQB1
164	Diisocyanate-induced Asthma	31898	4.4E-03	4.9E-02	5.0E+00	2.7E+01	NIN;SH3PXD2A;PRKCE;ZBTB16;PARD3B
165	Triglyceride Levels In Non-Type 2 Diabetes	8/209	4.4E-03	4.9E-02	3.3E+00	1.8E+01	ABCA1;MACF1;SCARB1;SEC16A;PEMT;UBE2E2;LPP;SSBP3

Supplementary Table 10: DNA methylation signals that overlapped with traits from the UK Biobank GWAS database, p< 0.05

No	Term	Overlap	P-value	Adjusted P-value	Old P-value	Old Adjusted P-value	Odds Ratio	Combined Score	Genes
1	Distance between home and job workplace 796 raw	88/3603	8.73E-11	2.5E-08	0	0	2.390006	55.35549	SCARB1;DGKD;MAST4;MEGF11;DOCK9;GOLIM4;CHD6; ADARB2;AFF3;PPP1R9A;SIPA1L3;BEST3;FARS2;SMG6;SYNE1; RPTOR;GHR;IMMP2L;IPO8;TIFAB;GRM4;MPRIP;SH3PXD2A; FAM3C;EGFL7;CAMK1D;VWF;KSR1;TRPC4;PRKCE;IL1R2;EBF1;GAB1;DDX10;UBE2E2;FRMD4A;NAV2;DUSP6;RUNX1;PARD3B;NCOR2;ZEB1;PCCA;HECW2;CDH13;RAB38;L3MBTL4;EXOC2; MGLL;MAD1L1;SHANK2;SDCCAG8;SHANK1;HDAC4;MACF1; ZCCHC7;FUT10;SEZ6;PRR5L;IQGAP2;JAKMIP1;LPP;PAPSS2; PDGFD;MGAT5;MAN1C1;ST8SIA5;CAMTA1;APBB2;LRRC8A; BRD8;IGF2BP2;NCAM2;WASF1;TRPM3;STARD13;RABGAP1L; ZBTB16;MX1;TRAPPC9;TBX4;HS3ST1;TMEM59;NT5C1A; CACHD1;SPATS2L;TCF4;NES
2	High light scatter reticulocyte percentage 30290 raw	100/4373	1.17E-10	2.5E-08	0	0	2.274089	51.99766	RXFP4;GOLIM4;JADE1;ADARB2;BEST3;EDC3;ZFYVE28;IPO8; NAMPT;PPFIA1;NEK2;FAM3C;CHGB;PRKCE;EBF1;DDX10; UBE2E2;FRMD4A;RUNX1;HECW2;SPATA13;RAB38;CARD11; MAD1L1;ZCCHC7;IQGAP2;ZBTB4;LPP;ADAMTS13;BAG5; PDGFD;CAMTA1;MYH10;TRPM3;ABCA1;STARD13;RABGAP1L;SLC10A7;NEK6;TRAPPC9;TRERF1;ZNF30;LHX5;TCF4;ZNF496; HIP1;MAST4;SLC44A2;DOCK9;LRRK2;CHD6;RCSD1;AFF3; PPP1R9A;FARS2;SMG6;GHR;IMMP2L;SLC22A18;ABCC1; CAMK1D;SYK;TRPC4;DNMT3A;NAV2;DUSP6;PARD3B;NCOR2;DMRTA2;ZEB1;HEATR5A;IRF2;CDH13;L3MBTL4;EXOC2;MGLL;SHANK2;SHANK1;HDAC4;SEZ6;PRR5L;JAKMIP1;NKD1;MGAT5;APBB2;LRRC8A;IGF2BP2;NCAM2;WASF1;DNAJC5B;GABRA5; ERAP1;SYT16;ZIC4;HS3ST1;NIN;PPP2R2C;WT1;NFIC;CACHD1
3	Monocyte count 30130 raw	52/1810	8.61E-09	1.22E-06	0	0	2.643078	49.08282	MOGAT2;LRRK2;GOLIM4;CHD6;ADARB2;RCSD1;AFF3;SMG6; IMMP2L;BZRAP1;GRM4;NAMPT;FAM3C;JAK1;CAMK1D;SYK; KSR1;PRKCE;EBF1;DDX10;NAV2;RUNX1;PARD3B;NCOR2; PACS2;CDH13;RAB38;L3MBTL4;SHANK2;HLA-DQB1;SDCCAG8; HDAC4;MACF1;FUT10;ABHD6;LPP;PDGFD;TTC21B;SPOCK2; CAMTA1;IGF2BP2;NCAM2;TRPM3;NKX2-3;ABCA1;NEK6; SYT16;ZIC4;MAPK12;HS3ST1;TCF4;SSBP3
4	Duration to first press of snap-button in each round 404 raw	41/1382	1.69E-07	1.8E-05	0	0	2.671501	41.66109	HIP1;DOCK9;CHD6;SLC2A2;CREBL2;ADARB2;LPP;PAPSS2; RPTOR;GHR;IPO8;PDGFD;MGAT5;GSG1L;CAMTA1;APBB2; NCAM2;TRPM3;CNNM4;SLC10A7;PRKCE;NEK6;EBF1;DDX10; UBE2E2;FRMD4A;TRAPPC9;NAV2;PARD3B;NCOR2;MYO1C; PCCA;WT1;IRF2;CACHD1;SPATS2L;CDH13;TCF4;L3MBTL4;

									SSBP3;SHANK2
5	Body fat percentage 23099 raw	23/538	2.63E-07	2.24E-05	0	0	3.792591	57.45556	MAST4;ZBTB16;SSR3;EBF1;CHD6;SYT16;SLC2A2;AFF3;ZBTB4;MYO19;TSKU;DUSP6;RUNX1;PARD3B;RPTOR;NCKIPSD;NIN;GRM4;CAMTA1;CDH13;SSBP3;MAD1L1;SDCCAG8
6	Trunk fat percentage 23127 raw	22/513	4.57E-07	3.25E-05	0	0	3.796067	55.41315	MAST4;TNFSF12;SSR3;RXFP4;EBF1;CHD6;SYT16;AFF3;ZBTB4;MYO19;DUSP6;RUNX1;PARD3B;RPTOR;NCKIPSD;GRM4;MGAT5;SPATS2L;CDH13;SSBP3;MAD1L1;SDCCAG8
7	Longest period of depression 4609 raw	27/767	1.09E-06	6.18E-05	0	0	3.112419	42.72415	MACF1;SCARB1;MAST4;CCDC126;GOLIM4;ADARB2;PPP1R9A;RAPGEFL1;SMG6;IMMP2L;TMEM246;MGAT5;CAMTA1;NCAM2;MYH10;TRPM3;CAMK1D;KSR1;GAB1;FRMD4A;VWA5B1;PARD3B;TMEM59;PCCA;IRF2;CDH13;AMPH
8	Pulse wave Arterial Stiffness index 21021 raw	80/3865	1.16E-06	6.18E-05	0	0	1.929286	26.3673	FAAH;NPFFR1;HIP1;NUMA1;MEGF11;DOCK9;LRRK1;GOLIM4;CHD6;ADARB2;RCSD1;AFF3;PPP1R9A;BEST3;FARS2;SYNE1;RPTOR;GHR;IMMP2L;IPO8;GRM4;SH3PXD2A;FAM3C;CHGB;ABCC1;MYBPC2;SYK;VWF;KSR1;TRPC4;PRKCE;EBF1;GAB1;DDX10;UBE2E2;FRMD4A;NAV2;VWA5B1;DUSP6;RUNX1;PARD3B;ALDH3A2;PACS2;PCCA;HECW2;SPATA13;IRF2;CDH13;RAB38;EXOC2;MAD1L1;SHANK2;HDAC4;ZCCHC7;EXD3;FUT10;PRR5L;LPP;PDGFD;MGAT5;GSG1L;CAMTA1;ZSWIM7;IGF2BP2;NCAM2;MYH10;TRPM3;ABCA1;SLC10A7;NEK6;IRX4;PLEKHA5;SYT16;ZIC4;TBX4;HS3ST1;SPATS2L;PEMT;TCF4;FBXL4
9	Frequency of needing morning drink of alcohol after heavy drinking session in last year 20412	47/1843	1.52E-06	7.19E-05	0	0	2.293082	30.72267	BARHL1;ZNF232;HDAC4;MAST4;PAPLN;CHD6;ADARB2;RCSD1;AFF3;JAKMIP1;LPP;PAPSS2;NKD1;IFI44L;LRRC31;SMG6;ZFYVE28;RPTOR;GHR;IMMP2L;IPO8;ZMYM5;MGAT5;GSG1L;NEK2;NCAM2;MYH10;ABCA1;EGFL7;CAMK1D;NEK6;IRX4;UBE2E2;ZIC4;TRAPPC9;NAV2;TRERF1;ANKHD1;PARD3B;NCOR2;ALDH3A2;CDH13;AMPH;TCF4;L3MBTL4;SSBP3;MAD1L1
10	Microalbumin in urine 30500 raw	41/1522	2.01E-06	8.56E-05	0	0	2.40541	31.55351	HDAC4;MACF1;MAST4;FUT10;ADARB2;LPP;RPTOR;IMMP2L;GRM4;PDGFD;PPFIA1;MAN1C1;IGF2BP2;NCAM2;ABCA1;RABGAP1L;TLE2;SLC10A7;SYK;GABRA5;PRKCE;EBF1;FRMD4A;ZIC4;NAV2;TRERF1;LRP2BP;CKAP4;HS3ST1;PARD3B;ZEB1;IRF2;CDH13;TCF4;FBXL4;APBA2;CARS2;SSBP3;LGR4;SHANK2;CAPS2
11	Inverse distance to the nearest major road 24012 raw	36/1276	3.21E-06	0.000124	0	0	2.503259	31.66731	HDAC4;ZCCHC7;EXD3;MAST4;MEGF11;FUT10;LRRK1;GATA6;LPP;TSKU;SYNE1;GHR;IPO8;SH3PXD2A;PDGFD;CAMTA1;APBB2;NCAM2;TRPM3;ABCA1;STARD13;SLC10A7;DNAJC5B;TRPC4;NEK6;EBF1;PLEKHA5;DDX10;FRMD4A;PARD3B;PCCA;HECW2;LHX5;TCF4;L3MBTL4;MAD1L1
12	Longest period of unenthusiasm/disinterest 5375 raw	33/1132	4.29E-06	0.000144	0	0	2.577037	31.85203	BLK;MACF1;EXD3;CCDC126;DOCK9;CHD6;PRR5L;RCSD1;LPP;NYNRIN;ZFYVE28;FADS3;IMMP2L;SH3PXD2A;MGAT5;CAMTA1;APBB2;FAM3C;IGF2BP2;NCAM2;TRPM3;CAMK1D;

									NEK6;DNMT3A;IRX4;UBE2E2;HS3ST1;RUNX1;PARD3B;PCCA; PTK7;CDH13;AMPH
13	Impedance of left leg 23108 raw	23/635	4.39E-06	0.000144	0	0	3.179486	39.22247	BLK;MACF1;EXD3;HIP1;PRKCE;TRADD;EBF1;UBE2E2;SLC2A2; ADARB2;AFF3;ITIH1;SMG6;SYNE1;PARD3B;NYNRIN;RPTOR; NCOR2;GHR;MPRIP;TCF4;MAD1L1;HLA-DQB1
14	Right 6mm regularity index 5161 raw	44/1808	1.11E-05	0.000337	0	0	2.165309	24.70645	BLK;HDAC4;HIP1;FUT10;GOLIM4;CHD6;PRR5L;ADARB2;AFF3;PPP 1R9A;PAPSS2;FARS2;SYNE1;FADS3;GHR;IMMP2L;RINL; GRM4;MGAT5;FAM3B;CAMTA1;APBB2;NCAM2;TRPM3; CAMK1D;VWF;KSR1;PRKCE;DDX10;FRMD4A;LGALS16; TRAPPC9;VWA5B1;TBX4;DUSP6;PLEKHA7;RUNX1;PARD3B; PCCA;IRF2;CDH13;AMPH;RAB38;SHANK2
15	Left arm fat percentage 23123 raw	19/497	1.44E-05	0.000399	0	0	3.33646	37.20389	MACF1;ZCCHC7;SSR3;IRX4;EBF1;SYT16;SLC2A2;AFF3;ZBTB4;MYO 19;TSKU;DUSP6;RUNX1;PARD3B;RPTOR;CAMTA1; CDH13;CARD11;MAD1L1
16	Lymphocyte count 30120 raw	55/2500	1.5E-05	0.000399	0	0	1.975415	21.94473	MAST4;CD81;MEGF11;GOLIM4;CHD6;ADARB2;AFF3; PPP1R9A;SMG6;EDC3;SYNE1;RPTOR;GHR;GOLGA3;IMMP2L; SH3PXD2A;FAM3C;CCR2;CAMK1D;DUSP1;PRKCE;DNMT3A; EBF1;DDX10;UBE2E2;FRMD4A;NAV2;PARD3B;NCOR2;PCCA; HECW2;SPATA13;CDH13;L3MBTL4;EXOC2;SHANK2;FUT10; JAKMIP1;LPP;NKD1;SLC5A5;PDGFD;MGAT5;GSG1L;CAMTA1;NCA M2;TRPM3;ABCA1;IRX4;PLEKHA5;TRAPPC9;PLEKHA7; HS3ST1;NIN;TCF4
17	Left arm fat-free mass 23125 raw	28/948	1.87E-05	0.000468	0	0	2.586549	28.16344	BLK;MACF1;SLC44A2;DOCK9;SLC2A2;ADARB2;AFF3;IFI44L; RPTOR;GHR;GRM4;MOV10L1;CAMTA1;IGF2BP2;NCAM2; RABGAP1L;NEK6;DNMT3A;UBE2E2;PARD3B;FAM220A; NCOR2;NIN;WT1;NFIC;SPATS2L;LHX5;TCF4
18	Weight 23098 raw	22/662	2.67E-05	0.000606	0	0	2.895502	30.49467	BLK;MACF1;RABGAP1L;MAST4;SYT16;SLC2A2;AFF3;MYO19; RAPGEFL1;DUSP6;FARS2;PARD3B;FAM220A;RPTOR;NCOR2; GHR;GRM4;NFIC;CDH13;IGF2BP2;MAD1L1;HLA-DQB1
19	Waist circumference 48 raw	17/432	2.82E-05	0.000606	0	0	3.424578	35.87091	MACF1;ZCCHC7;MAST4;SSR3;SYT16;SLC2A2;AFF3;MYO19;DUSP6 ;PARD3B;FAM220A;RPTOR;NIN;CAMTA1;CDH13;MAD1L1;SDCCA G8
20	Hip circumference 49 raw	19/523	2.89E-05	0.000606	0	0	3.161159	33.03808	BLK;MACF1;RABGAP1L;MAST4;SYT16;SLC2A2;MYO19; RAPGEFL1;DUSP6;RUNX1;PARD3B;FAM220A;RPTOR;GOLGA3;GR M4;PEMT;CDH13;IGF2BP2;MAD1L1
21	Weight 21002 raw	22/667	2.99E-05	0.000606	0	0	2.872497	29.928	BLK;MACF1;RABGAP1L;MAST4;SYT16;SLC2A2;AFF3;MYO19; RAPGEFL1;DUSP6;FARS2;PARD3B;FAM220A;RPTOR;NCOR2; GHR;GRM4;NFIC;CDH13;IGF2BP2;MAD1L1;HLA-DQB1
22	Whole body fat mass 23100 raw	19/528	3.29E-05	0.000637	0	0	3.1295	32.30417	BLK;MACF1;RABGAP1L;MAST4;EBF1;SYT16;SLC2A2;AFF3; MYO19;DUSP6;PARD3B;FAM220A;RPTOR;GRM4;CAMTA1; CDH13;SSBP3;MAD1L1;HLA-DQB1
23	Right arm fat mass 23120 raw	17/443	3.87E-05	0.000716	0	0	3.334734	33.88135	BLK;MACF1;ZCCHC7;IRX4;SYT16;SLC2A2;AFF3;MYO19;TSKU; DUSP6;PARD3B;FAM220A;RPTOR;GRM4;CAMTA1;MAD1L1;

									HLA-DQB1
24	Right arm fat-free mass 23121 raw	24/788	4.64E-05	0.000823	0	0	2.650666	26.45121	BLK;MACF1;DOCK9;NEK6;LRRK1;DNMT3A;SLC2A2;FRMD4A; AFF3;IFI44L;PARD3B;FAM220A;RPTOR;NCOR2;GHR;GOLGA3;NIN; GRM4;PDGFD;NFIC;ZNF30;SPATS2L;TCF4;IGF2BP2
25	Trunk fat mass 23128 raw	19/559	7.04E-05	0.001199	0	0	2.946302	28.17255	MACF1;MAST4;EBF1;SYT16;SLC2A2;AFF3;MYO19;RAPGEFL1;DUS P6;PARD3B;FAM220A;RPTOR;NCKIPSD;GRM4;NFIC;CDH13;SSBP3 ;MAD1L1;HLA-DQB1
26	Left leg fat-free mass 23117 raw	22/715	8.3E-05	0.001329	0	0	2.66854	25.07594	BLK;MACF1;EXD3;DOCK9;DNMT3A;SLC2A2;AFF3;MYO19; RAPGEFL1;DUSP6;PARD3B;FAM220A;RPTOR;NCOR2;GHR; GOLGA3;GRM4;NFIC;IGF2BP2;SSBP3;MAD1L1;HLA-DQB1
27	Right leg fat mass 23112 raw	17/472	8.42E-05	0.001329	0	0	3.118695	29.25913	BLK;ZCCHC7;RABGAP1L;SYT16;SLC2A2;AFF3;MYO19;TSKU; DUSP6;PARD3B;FAM220A;RPTOR;GRM4;CAMTA1;CDH13; MAD1L1;HLA-DQB1
28	Left leg predicted mass 23118 raw	22/718	8.81E-05	0.001341	0	0	2.656727	24.80464	BLK;MACF1;EXD3;DOCK9;DNMT3A;SLC2A2;AFF3;MYO19; RAPGEFL1;DUSP6;PARD3B;FAM220A;RPTOR;NCOR2;GHR; GOLGA3;GRM4;NFIC;IGF2BP2;SSBP3;MAD1L1;HLA-DQB1
29	Right arm fat percentage 23119 raw	17/477	9.57E-05	0.001377	0	0	3.084201	28.54302	BLK;MACF1;ZCCHC7;IRX4;EBF1;SYT16;SLC2A2;AFF3;ZBTB4; TSKU;DUSP6;PARD3B;FAM220A;RPTOR;CAMTA1;CARD11; MAD1L1
30	Impedance of right leg 23107 raw	20/622	9.7E-05	0.001377	0	0	2.784313	25.73057	BLK;MACF1;EXD3;HIP1;TRADD;EBF1;UBE2E2;SLC2A2; ADARB2;AFF3;DUSP6;SMG6;NYNRIN;RPTOR;NCOR2;GHR; MPRIP;TCF4;MAD1L1;HLA-DQB1
31	Body mass index (BMI) 21001 raw	19/580	0.000114	0.001563	0	0	2.833704	25.7343	BLK;ZCCHC7;RABGAP1L;SYT16;SLC2A2;AFF3;LPP;TSKU; DUSP6;PARD3B;FAM220A;RPTOR;CDH13;TCF4;TRPM3; CARD11;MAD1L1;HLA-DQB1;SDCCAG8
32	Platelet crit 30090 raw	20/635	0.000128	0.001656	0	0	2.72408	24.42036	MACF1;CSRNP1;DGKD;MAST4;VWF;DNAJC5B;PRKCE;TNFSF12;ZB TB16;PRR5L;IQGAP2;AFF3;RUNX1;SMG6;BZRAP1;NFIC;PEMT;TCF 4;IGF2BP2;NKX2-3
33	Diastolic blood pressure (automated reading) 4079 raw	13/310	0.000132	0.001656	0	0	3.629093	32.42572	ZCCHC7;EXD3;EGFL7;TRPC4;PRKCE;EBF1;SMG6;LFNG;NCOR2;NIN ;CDH13;SDCCAG8;HLA-DQB1
34	Left leg fat percentage 23115 raw	18/538	0.000132	0.001656	0	0	2.891449	25.82041	ZBTB16;EBF1;CHD6;SYT16;UBE2E2;SLC2A2;AFF3;TSKU; DUSP6;RUNX1;PARD3B;RPTOR;NCKIPSD;NIN;CAMTA1; CDH13;MAD1L1;SDCCAG8
35	Left leg fat mass 23116 raw	17/492	0.000139	0.001656	0	0	2.985073	26.52006	ZCCHC7;RABGAP1L;MAST4;SYT16;SLC2A2;AFF3;MYO19; TSKU;DUSP6;PARD3B;FAM220A;RPTOR;GRM4;CAMTA1; CDH13;MAD1L1;HLA-DQB1
36	Whole body water mass 23102 raw	24/847	0.00014	0.001656	0	0	2.454964	21.78557	BLK;MACF1;SLC44A2;DUSP1;MEGF11;DOCK9;NEK6;DNMT3A;SLC 2A2;AFF3;PARD3B;FAM220A;RPTOR;NCOR2;GHR; GOLGA3;GRM4;NFIC;CAMTA1;TCF4;IGF2BP2;SSBP3;MAD1L1;HL A-DQB1

37	Monocyte percentage 30190 raw	13/321	0.000185	0.002136	0	0	3.498004	30.05651	SCARB1;KSR1;NEK6;ZBTB16;EBF1;LPP;RUNX1;GHR;IMMP2L;BZRAP1;AMPH;NKX2-3;CCR2
38	Body mass index (BMI) 23104 raw	18/555	0.000194	0.002174	0	0	2.798069	23.91827	BLK;ZCCHC7;RABGAP1L;SYT16;SLC2A2;AFF3;LPP;MYO19;TSKU;DUSP6;PARD3B;RPTOR;CDH13;TCF4;TRPM3;CARD11;MAD1L1;HLA-DQB1
39	Haematocrit percentage 30030 raw	15/415	0.00021	0.002297	0	0	3.116707	26.38883	EXD3;STARD13;RABGAP1L;MOGAT2;PRKCE;UBE2E2;FRMD4A;VWA5B1;RUNX1;LFNG;NCOR2;MPRIP;PTK7;RANBP10;SFTPA1
40	Left arm fat mass 23124 raw	15/422	0.000251	0.002677	0	0	3.062276	25.38094	BLK;MACF1;ZCCHC7;SYT16;SLC2A2;AFF3;MYO19;DUSP6;PARD3B;FAM220A;RPTOR;GRM4;CAMTA1;MAD1L1;HLA-DQB1
41	Platelet count 30080 raw	21/722	0.000258	0.002677	0	0	2.509161	20.73511	BLK;CSRNP1;DGKD;SYK;MAST4;DNAJC5B;PRKCE;ZBTB16;LRRK2;ERAP1;EBF1;SEZ6;IQGAP2;RUNX1;SMG6;BZRAP1;NFIC;CAMTA1;TCF4;IGF2BP2;NKX2-3
42	Reticulocyte percentage 30240 raw	31/1284	0.000276	0.002795	0	0	2.096099	17.18115	HDAC4;ZCCHC7;MAST4;SLC44A2;GOLIM4;CHD6;ADARB2;RCSD1;AFF3;ZFYVE28;IPO8;PDGFD;NAMPT;IGF2BP2;NCAM2;STARD13;RABGAP1L;DNAJC5B;TRPC4;PRKCE;EBF1;SYT16;UBE2E2;FRMD4A;NAV2;TBX4;DUSP6;RUNX1;PCCA;SPATA13;AMPH
43	Impedance of whole body 23106 raw	21/730	0.000298	0.002955	0	0	2.480074	20.13175	FLG;BLK;MACF1;EXD3;HIP1;NEK6;EBF1;SLC2A2;ADARB2;AFF3;FARS2;SMG6;PARD3B;NYNRIN;RPTOR;NCOR2;GHR;SH3PXD2A;SERPINH1;TCF4;TRPM3
44	Comparative height size (age 10) 1697	21/742	0.00037	0.003459	0	0	2.437655	19.26401	ABCC1;TNFSF12;DNMT3A;SIPA1L3;LPP;PLEKHA7;NCOR2;NCKIPSD;GHR;DMRTA2;ADAMTS13;SH3PXD2A;NFIC;SPOCK2;SERPINH1;TCF4;IGF2BP2;SSBP3;CNNM4;TRIM23;HLA-DQB1
45	Heel bone mineral density 3148 raw	13/345	0.000371	0.003459	0	0	3.242144	25.60864	BLK;DOCK9;PRKCE;DNMT3A;EBF1;ZBTB4;TBX4;RUNX1;SMG6;SYNE1;GHR;NFIC;FAM3C
46	Whole body fat-free mass 23101 raw	23/853	0.000388	0.003459	0	0	2.32452	18.25898	MACF1;SLC44A2;DUSP1;MEGF11;DOCK9;NEK6;DNMT3A;SLC2A2;AFF3;PARD3B;FAM220A;RPTOR;NCOR2;GHR;GOLGA3;GRM4;NFIC;CAMTA1;TCF4;IGF2BP2;SSBP3;MAD1L1;HLA-DQB1
47	Right leg fat-free mass 23113 raw	21/745	0.00039	0.003459	0	0	2.42727	19.05379	MACF1;EXD3;DOCK9;DNMT3A;SLC2A2;AFF3;RAPGEFL1;DUSP6;PARD3B;RPTOR;NCOR2;GHR;GOLGA3;GRM4;NFIC;SPATS2L;PEMT;IGF2BP2;SSBP3;MAD1L1;HLA-DQB1
48	Right leg predicted mass 23114 raw	21/745	0.00039	0.003459	0	0	2.42727	19.05379	MACF1;EXD3;DOCK9;DNMT3A;SLC2A2;AFF3;RAPGEFL1;DUSP6;PARD3B;RPTOR;NCOR2;GHR;GOLGA3;GRM4;NFIC;SPATS2L;PEMT;IGF2BP2;SSBP3;MAD1L1;HLA-DQB1
49	Time spent doing light physical activity 104920	17/543	0.000435	0.003779	0	0	2.690329	20.82593	HIP1;IRX4;TSKU;HS3ST1;PARD3B;ZFYVE28;FADS3;IMMP2L;IPO8;NIN;ZEB1;SH3PXD2A;CDH13;APBB2;AMPH;NCAM2;EXOC2

50	Basal metabolic rate 23105 raw	22/812	0.000485	0.004136	0	0	2.332027	17.79419	BLK;MACF1;SLC44A2;MEGF11;DOCK9;DNMT3A;SLC2A2;AFF3;PARD3B;FAM220A;RPTOR;NCOR2;GHR;GOLGA3;GRM4;NFIC;CAMTA1;TCF4;IGF2BP2;SSBP3;MAD1L1;HLA-DQB1
51	Mean platelet volume 30100 raw	20/711	0.000548	0.004578	0	0	2.417306	18.15162	ZNF496;SYK;NUMA1;MAST4;ZBTB16;LRRK2;EBF1;SEZ6; KLHL12;IQGAP2;AFF3;RUNX1;NCOR2;MYO1C;MPRIP;PEMT; CAMTA1;LRRC8A;LDLRAP1;TRPM3
52	Trunk predicted mass 23130 raw	23/878	0.000577	0.004725	0	0	2.25434	16.81328	BLK;MACF1;SLC44A2;DUSP1;DOCK9;NEK6;LRRK1;SLC2A2; PARD3B;FAM220A;RPTOR;NCOR2;GHR;GOLGA3;DMRTA2; NIN;GRM4;NFIC;MOV10L1;SPATS2L;CAMTA1;TCF4;IGF2BP2
53	Ankle spacing width 3143 raw	14/414	0.000655	0.005268	0	0	2.899633	21.25518	ABCA1;BLK;DUSP1;TNFSF12;EBF1;AFF3;SMG6;NCOR2;GHR; DMRTA2;HECW2;TCF4;KIFAP3;HLA-DQB1
54	Right leg fat percentage 23111 raw	16/515	0.000691	0.005364	0	0	2.663282	19.38317	ZBTB16;EBF1;SYT16;UBE2E2;SLC2A2;AFF3;TSKU;DUSP6; RUNX1;PARD3B;RPTOR;NIN;CAMTA1;CDH13;MAD1L1; SDCCAG8
55	Pulse wave reflection index 4195 raw	21/779	0.000692	0.005364	0	0	2.315316	16.84443	STARD13;SYK;FUT10;NEK6;GATA6;UBE2E2;SLC2A2;ABHD6; ZIC4;TRAPPC9;SIPA1L3;HS3ST1;PARD3B;IPO8;WT1;NFIC; CDH13;RAB38;TCF4;NCAM2;APBA2
56	Standing height 50 raw	32/1420	0.000716	0.005446	0	0	1.949338	14.11703	CUL7;DOCK9;PAPLN;SEZ6;GOLIM4;ADARB2;AFF3;PPP1R9A; ZBTB4;SIPA1L3;LPP;IFI44L;SH3PXD2A;SERPINH1;IGF2BP2; CNNM4;TRIM23;JAK1;STARD13;RABGAP1L;ABCC1;DUSP1; DNMT3A;EBF1;UBE2E2;ZIC4;CKAP4;NCOR2;DMRTA2;NFIC; TCF4;SSBP3
57	Doctor diagnosed hayfever/allergic rhinitis/eczema 6152 9	8/161	0.000872	0.006517	0	0	4.29086	30.2279	FLG;NEK6;DNMT3A;UBE2E2;PEMT;LPP;CARD11;HLA-DQB1
58	ICD10 malignant neoplasm of breast C50	42430	0.00091	0.006681	0	0	18.74501	131.2613	IPO8;EBF1;SYNE1
59	Heel Broadband ultrasound attenuation, direct entry 3144 raw	11/295	0.001123	0.008109	0	0	3.192603	21.68299	BLK;GHR;MACF1;DOCK9;PRKCE;NFIC;SPATA13;EBF1;CHD6; RUNX1;SMG6
60	Basophill percentage 30220 raw	9/214	0.001375	0.009763	0	0	3.606903	23.76692	HIP1;MYO1C;ESRP2;ERAP1;CDH13;NAV2;TRPM3;RASGRP4; SHANK2
61	Other exercises in last 4 wks 6164 2	43525	0.001532	0.010674	0	0	15.22859	98.70222	IRF2;AFF3;MAD1L1
62	Overall health rating 2178	6/101	0.001553	0.010674	0	0	5.162027	33.38418	SSR3;TCF4;TSKU;MAD1L1;DUSP6;SDCCAG8

63	Trunk fat-free mass 23129 raw	22/890	0.001584	0.010711	0	0	2.115985	13.64339	BLK;MACF1;SLC44A2;DUSP1;DOCK9;NEK6;DNMT3A;SLC2A2; IFI44L;PARD3B;FAM220A;RPTOR;NCOR2;GHR;GOLGA3; GRM4;NFIC;MOV10L1;SPATS2L;CAMTA1;TCF4;IGF2BP2
64	None of the above pain types in last month 6159 100	43891	0.001786	0.011886	0	0	14.33224	90.69341	MACF1;CAMK1D;TSKU
65	Takes none of the above medications 6154 100	15797	0.001882	0.012332	0	0	8.348654	52.39281	RABGAP1L;TCF4;MAD1L1;HLA-DQB1
66	Right arm predicted mass 23122 raw	20/792	0.001999	0.012904	0	0	2.156841	13.40483	BLK;MACF1;DOCK9;NEK6;LRRK1;DNMT3A;SLC2A2;FRMD4A; AFF3;IFI44L;PARD3B;FAM220A;NCOR2;GHR;GRM4;PDGFD; NFIC;SPATS2L;TCF4;IGF2BP2
67	Home large urban area scotland 20118 11	8/186	0.002181	0.013869	0	0	3.684689	22.57914	NCOR2;ABCC1;IL1R2;LRRK2;UBE2E2;CDH13;AFF3;JAKMIP1
68	Left arm predicted mass 23126 raw	19/743	0.002236	0.014006	0	0	2.181841	13.3162	BLK;MACF1;RABGAP1L;SLC44A2;DOCK9;NEK6;DNMT3A; SLC2A2;PARD3B;FAM220A;RPTOR;NCOR2;GHR;NIN;GRM4; NFIC;CAMTA1;TCF4;IGF2BP2
69	Hair/balding pattern 4 2395 4	8/192	0.002654	0.015974	0	0	3.563718	21.13896	TRADD;SOX13;EBF1;TCF4;EXOC2;LGR4;RUNX1;SMG6
70	No surgery/amputatio n of toe/leg 5540 0	16/588	0.002672	0.015974	0	0	2.316821	13.72705	NPFFR1;VWF;DOCK9;JADE1;LGALS16;TTF2;AFF3;RPTOR;IMMP2L; IPO8;PDGFD;SPATS2L;CAMTA1;TCF4;NCAM2;SHANK2
71	Malignant neoplasm of breast C3 BREAST 3	44986	0.0027	0.015974	0	0	12.18102	72.04572	EBF1;CDH13;SYNE1
72	Malignant neoplasm of breast C BREAST 3	44986	0.0027	0.015974	0	0	12.18102	72.04572	EBF1;CDH13;SYNE1
73	Miserableness 1930	17624	0.002828	0.016503	0	0	7.398536	43.41596	BLK;RABGAP1L;TCF4;MAD1L1
74	Diabetes related eye disease 6148 1	45109	0.003024	0.017411	0	0	32.40123	187.96	IGF2BP2;HLA-DQB1
75	Age asthma diagnosed 3786 raw	45717	0.003443	0.019301	0	0	11.07281	62.7974	FLG;LPP;HLA-DQB1
76	Drive faster than motorway speed limit 1100	45717	0.003443	0.019301	0	0	11.07281	62.7974	DOCK9;NFIC;TCF4
77	Rarely/never worked with asbestos 22612 0	7/162	0.003962	0.0213	0	0	3.694194	20.43279	MPRIP;DOCK9;MGAT5;IRX4;GOLIM4;PPP1R9A;EXOC2

78	Cake intake 102190	45140	0.004	0.0213	0	0	27	149.0792	CDH13;BEST3
79	Leisure/social none of the above 6160 100	45140	0.004	0.0213	0	0	27	149.0792	AFF3;MAD1L1
80	Physically abused by family as a child 20488	45140	0.004	0.0213	0	0	27	149.0792	DCLRE1C;RAMP1
81	Heel bone mineral density T-score 78 raw	11/349	0.004123	0.021419	0	0	2.676981	14.69987	BLK;GHR;DOCK9;PRKCE;NFIC;DNMT3A;EBF1;ZBTB4;RUNX1; SMG6;SYNE1
82	Heel quantitative ultrasound index direct entry 3147 raw	11/349	0.004123	0.021419	0	0	2.676981	14.69987	BLK;GHR;DOCK9;PRKCE;NFIC;DNMT3A;EBF1;ZBTB4;RUNX1; SMG6;SYNE1
83	Number of self-reported non-cancer illnesses 135	46447	0.004301	0.02181	0	0	10.14931	55.30354	EBF1;MAD1L1;HLA-DQB1
84	Venous thromboembolism I9 VTE	46447	0.004301	0.02181	0	0	10.14931	55.30354	SLC44A2;ADARB2;KIFAP3
85	College/university degree 6138 1	10/307	0.004874	0.024426	0	0	2.765191	14.7216	CAMK1D;PCCA;CAMTA1;CDH13;RAB38;TCF4;AFF3;AJUBA; MAD1L1;SDCCAG8
86	Vitamin C intake 100015 raw	32994	0.005061	0.024979	0	0	4.794666	25.34594	ZFYVE28;BLK;RINL;CDH13;TSKU
87	Self-reported uterine fibroids 20002 1351	45171	0.005101	0.024979	0	0	23.14198	122.1491	WT1;SYNE1
88	Hair/balding pattern 3 2395 3	20911	0.005268	0.025215	0	0	6.140078	32.21148	PCCA;EBF1;LGR4;RUNX1
89	Lifetime number of sexual partners 2149	20911	0.005268	0.025215	0	0	6.140078	32.21148	BLK;JADE1;TCF4;MAD1L1
90	Red blood cell count 30010 raw	15/576	0.005344	0.025296	0	0	2.208453	11.55406	BLK;STARD13;MOGAT2;DNAJC5B;PRKCE;ZBTB16;TRADD; UBE2E2;FRMD4A;IQGAP2;AFF3;LPP;RUNX1;TUBGCP4;NKX2-3
91	Spirits intake 100730	21276	0.005605	0.026237	0	0	6.026144	31.24059	DMRTA2;CAMTA1;RAB38;PARD3B
92	Workplace rarely/never had diesel exhaust 22615 0	6/133	0.006077	0.028139	0	0	3.856649	19.68139	SH3PXD2A;TRPC4;FUT10;PLEKHA5;ZIC4;SMG6

93	None of the above (1) in siblings 20111 100	45201	0.006325	0.028974	0	0	20.24846	102.5222	EBF1;HLA-DQB1
94	Age first had sexual intercourse 2139 raw	6/136	0.006757	0.030624	0	0	3.767218	18.8252	BLK;HIP1;SPATS2L;TCF4;AFF3;MAD1L1
95	Sleep duration 1160	23102	0.007503	0.033644	0	0	5.514404	26.97907	BLK;ZCCHC7;TCF4;MAD1L1
96	ICD10 leiomyoma of uterus D25	45232	0.007669	0.034029	0	0	17.99795	87.66137	WT1;SYNE1
97	Reticulocyte count 30250 raw	20/908	0.008885	0.039019	0	0	1.866582	8.816699	RABGAP1L;MAST4;SLC44A2;DNAJC5B;PRKCE;CHD6;SYT16; NAV2;ADARB2;IQGAP2;AFF3;FARS2;BAG5;PTK7;NAMPT; SPATA13;CAMTA1;APBB2;IGF2BP2;NCAM2
98	Frequency of feeling guilt or remorse after drinking alcohol in last year 20409	45262	0.009128	0.039381	0	0	16.19754	76.07006	SPOCK2;MYH10
99	Longest period spent worried or anxious 20420 raw	9/286	0.009152	0.039381	0	0	2.662012	12.49493	RPTOR;MACF1;CDH13;APBB2;FRMD4A;ZIC4;NAV2;WASF1; SHANK2
100	Worrier/anxious feelings 1980	24563	0.009294	0.039594	0	0	5.163497	24.15665	BLK;CAMTA1;TCF4;HLA-DQB1
101	Ischaemic heart disease wide definition I9 IHD	13940	0.011235	0.047386	0	0	6.956614	31.22652	PDGFD;CDH13;SMG6

Supplementary Table 11: EWAS Catalog lookup for significant *APOL1* nephropathy-associated CpGs

Author	PMID	Trait	Analysis	CpG	Chrom Pos	Gene	Effect Estimate	SE	P value
Robert F Hillary	37410739	Prevalent Chronic Kidney Disease (Estimated)	Basic model	cg22959742	chr10:13913931	FRMD4A	0.074	0.01239	2E-09
Chu A.	29097680	Chronic kidney disease	Meta-analysis of prevalent CKD EWAS	cg22959742	chr10:13913931	FRMD4A	0.432	0.0905	1.9E-06
Robert F Hillary	37410739	Prevalent Chronic Kidney Disease (Estimated)	Fully-adjusted Model	cg22959742	chr10:13913931	FRMD4A	0.06	0.0128	3.2E-06
Chu A.	29097680	Chronic kidney disease	Meta-analysis of prevalent CKD EWAS	cg24791666	chr3:39192215	CSRNP1	-0.043	0.0073	3.8E-09
Robert F Hillary	37410739	Prevalent Chronic Kidney Disease (Estimated)	Basic model	cg01936957	chr12:124982370	NCOR2	0.12	0.02572	2.5E-06

Supplementary Table 12A: EWAS catalog lookup results for CKD-associated methylation signals at gene level (Blood pressure)

Author	PMID	Trait	Analysis	Cpg*	Chromosome	Gene	Effect estimate	p value
Richard MA	29198723	Diastolic Blood Pressure	Meta-analysis	cg09001549	12	SLC15A4	1.10E-07	8.70E-08
Richard MA	29198723	Systolic Blood Pressure	Meta-analysis	cg09001549	12	SLC15A4	-5.80E-05	7.10E-06
Richard MA	29198723	Diastolic Blood Pressure	Meta-analysis	cg19693031	1	TXNIP	-3.00E-04	1.80E-14
Huang D	32520614	Diastolic Blood Pressure	Discovery	cg19693031	1	TXNIP	NA	4.60E-05
Richard MA	29198723	Systolic Blood Pressure	Meta-analysis	cg19693031	1	TXNIP	-2.00E-04	3.10E-29
Richard MA	29198723	Systolic Blood Pressure	Meta-analysis	cg19693031	1	TXNIP	-2.00E-04	3.10E-29
Huang D	32520614	Systolic Blood Pressure	Discovery	cg19693031	1	TXNIP	NA	2.20E-07
Richard MA	29198723	Systolic Blood Pressure	Meta-analysis	cg24960291	3	IGF2BP2	-9.50E-05	6.00E-08

Supplementary Table 12B: EWAS catalog lookup results for CKD-associated methylation signals at gene level (CKD)

**Author**	**PMID**	**Trait**	**Analysis**	**Cpg***	**Chrom**	**Gene**	**Effect estimate**	**Standard Error**	**p value**
Chu A.	29097680	Chronic kidney disease	Meta-analysis of estimated glomerular filtration rate EWAS	cg10118456	16	ABCC1	-0.026	5.80E-03	8.10E-06
Chu A.	29097680	Chronic kidney disease	Meta-analysis of estimated glomerular filtration rate EWAS	cg23889047	1	CAMTA1	-0.033	6.80E-03	7.90E-07
Chu A.	29097680	Chronic kidney disease	Meta-analysis of prevalent CKD EWAS	cg00501876	3	CSRNP1	-0.044	5.60E-03	3.60E-15
Chu A.	29097680	Chronic kidney disease	Meta-analysis of prevalent CKD EWAS	cg03540589	3	CSRNP1	-0.022	4.40E-03	4.20E-07
Chu A.	29097680	Chronic kidney disease	Meta-analysis of prevalent CKD EWAS	cg24791666	3	CSRNP1	-0.043	7.30E-03	3.80E-09
Chu A.	29097680	Chronic kidney disease	Meta-analysis of prevalent CKD EWAS	cg22959742	10	FRMD4A	0.432	9.05E-02	1.90E-06
Chu A.	29097680	Chronic kidney disease	Meta-analysis of prevalent CKD EWAS	cg13085627	15	LRRK1	-0.043	8.70E-03	6.50E-07
Chu A.	29097680	Chronic kidney disease	Meta-analysis of estimated glomerular filtration rate EWAS	cg05417607	17	MYO1C	0.023	4.20E-03	4.80E-08
Chu A.	29097680	Chronic kidney disease	Meta-analysis of estimated glomerular filtration rate EWAS	cg22820108	12	NCOR2	-0.043	8.70E-03	8.40E-07
Chu A.	29097680	Chronic kidney disease	Meta-analysis of estimated glomerular filtration rate EWAS	cg06937357	14	PACS2	0.021	4.80E-03	9.90E-06
Chu A.	29097680	Chronic kidney disease	Meta-analysis of prevalent CKD EWAS	cg06937357	14	PACS2	-0.334	7.39E-02	6.30E-06
Chu A.	29097680	Chronic kidney disease	Meta-analysis of incident CKD EWAS	cg22908922	14	PACS2	0.29	6.00E-02	9.90E-07

Chu A.	29097680	Chronic kidney disease	Meta-analysis of estimated glomerular filtration rate EWAS	cg11704631	21	RUNX1	-0.025	5.10E-03	1.10E-06
Chu A.	29097680	Chronic kidney disease	Meta-analysis of estimated glomerular filtration rate EWAS	cg04049253	11	ZBTB16	-0.028	6.30E-03	7.20E-06
Robert F Hillary	37410739	Prevalent Chronic Kidney Disease (Estimated)	Basic model	cg00013899	1	CACHD1	0.051	9.48E-03	8.10E-08
Robert F Hillary	37410739	Prevalent Chronic Kidney Disease (Estimated)	Fully-adjusted Model	cg00013899	1	CACHD1	0.04	9.87E-03	4.20E-05
Robert F Hillary	37410739	Incident Chronic Kidney Disease	Fully-adjusted model	cg00067518	1	CAMTA1	-0.018	4.27E-03	4.10E-05
Robert F Hillary	37410739	Incident Chronic Kidney Disease	Basic model	cg00812921	19	GPR4	0.015	3.66E-03	2.80E-05
Robert F Hillary	37410739	Prevalent Chronic Kidney Disease (Estimated)	Basic model	cg01816454	9	LRRC8A	-0.062	1.53E-02	4.50E-05
Robert F Hillary	37410739	Prevalent Chronic Kidney Disease (Estimated)	Basic model	cg01936957	12	NCOR2	0.12	2.57E-02	2.50E-06
Robert F Hillary	37410739	Incident Chronic Kidney Disease	Basic model	cg02030654	10	FRMD4A	0.026	6.57E-03	7.10E-05
Robert F Hillary	37410739	Incident Chronic Kidney Disease	Fully-adjusted model	cg02069674	16	CDH13	-0.01	2.63E-03	7.00E-05
Robert F Hillary	37410739	Prevalent Chronic Kidney Disease (Estimated)	Fully-adjusted Model	cg02390329	3	ZIC4	0.035	8.49E-03	3.70E-05
Robert F Hillary	37410739	Incident Chronic Kidney Disease	Basic model	cg02514318	2	HECW2	-0.018	4.13E-03	1.20E-05
Robert F Hillary	37410739	Incident Chronic Kidney Disease	Basic model	cg03624563	5	ERAP1	0.0076	1.83E-03	3.30E-05
Robert F Hillary	37410739	Incident Chronic Kidney Disease	Basic model	cg03723730	6	GRM4	0.024	5.52E-03	2.00E-05
Robert F Hillary	37410739	Incident Chronic Kidney Disease	Fully-adjusted model	cg03723730	6	GRM4	0.023	5.91E-03	8.60E-05
Robert F Hillary	37410739	Prevalent Chronic Kidney Disease (Estimated)	Basic model	cg03778809	8	CLN8	0.046	1.13E-02	4.80E-05
Robert F Hillary	37410739	Prevalent Chronic Kidney Disease (Estimated)	Basic model	cg03877706	21	NCAM2	-0.027	6.49E-03	2.90E-05
Robert F Hillary	37410739	Prevalent Chronic Kidney Disease (Estimated)	Basic model	cg04819977	2	CNNM4	0.068	1.65E-02	4.40E-05
Robert F Hillary	37410739	Prevalent Chronic Kidney Disease (Estimated)	Basic model	cg05165263	4	IRF2	0.053	9.13E-03	4.90E-09

Robert F Hillary	37410739	Prevalent Chronic Kidney Disease (Estimated)	Fully-adjusted Model	cg05165263	4	IRF2	0.039	9.41E-03	3.70E-05
Robert F Hillary	37410739	Incident Chronic Kidney Disease	Basic model	cg05612693	21	RUNX1	-0.016	3.88E-03	4.00E-05
Robert F Hillary	37410739	Prevalent Chronic Kidney Disease (Estimated)	Basic model	cg06637924	10	MAT1A	0.068	1.55E-02	1.10E-05
Robert F Hillary	37410739	Prevalent Chronic Kidney Disease (Estimated)	Basic model	cg08752726	21	RUNX1	-0.044	1.12E-02	8.60E-05
Robert F Hillary	37410739	Prevalent Chronic Kidney Disease (Estimated)	Basic model	cg08791347	10	FRMD4A	0.044	1.06E-02	3.20E-05
Robert F Hillary	37410739	Prevalent Chronic Kidney Disease (Estimated)	Basic model	cg09377531	8	TRAPPC9	-0.03	7.25E-03	2.70E-05
Robert F Hillary	37410739	Prevalent Chronic Kidney Disease (Estimated)	Basic model	cg11698333	5	EBF1	0.072	1.61E-02	6.40E-06
Robert F Hillary	37410739	Prevalent Chronic Kidney Disease (Estimated)	Fully-adjusted Model	cg11698333	5	EBF1	0.069	1.63E-02	2.60E-05
Robert F Hillary	37410739	Prevalent Chronic Kidney Disease (Estimated)	Basic model	cg11704631	21	RUNX1	0.055	1.42E-02	9.90E-05
Robert F Hillary	37410739	Incident Chronic Kidney Disease	Basic model	cg11853678	9	ZCCHC7	0.016	3.54E-03	6.30E-06
Robert F Hillary	37410739	Prevalent Chronic Kidney Disease (Estimated)	Basic model	cg12450789	9	LRRC8A	-0.045	1.10E-02	5.40E-05
Robert F Hillary	37410739	Prevalent Chronic Kidney Disease (Estimated)	Basic model	cg13085627	15	LRRK1	0.071	1.63E-02	1.40E-05
Robert F Hillary	37410739	Prevalent Chronic Kidney Disease (Estimated)	Fully-adjusted Model	cg13789229	1	CAMTA1	0.067	1.61E-02	3.40E-05
Robert F Hillary	37410739	Incident Chronic Kidney Disease	Basic model	cg14721232	4	PPP2R2C	0.021	4.61E-03	3.80E-06
Robert F Hillary	37410739	Incident Chronic Kidney Disease	Basic model	cg15058210	2	HDAC4	-0.015	3.19E-03	4.90E-06
Robert F Hillary	37410739	Prevalent Chronic Kidney Disease (Estimated)	Basic model	cg15659943	9	ABCA1	0.082	1.83E-02	6.80E-06
Robert F Hillary	37410739	Incident Chronic Kidney Disease	Basic model	cg17470549	16	CDH13	-0.013	2.34E-03	9.20E-08
Robert F Hillary	37410739	Prevalent Chronic Kidney Disease (Estimated)	Basic model	cg18001454	11	PPFIA1	0.047	1.19E-02	7.90E-05
Robert F Hillary	37410739	Incident Chronic Kidney Disease	Basic model	cg20124610	13	CARS2	-0.021	4.77E-03	1.40E-05

Robert F Hillary	37410739	Incident Chronic Kidney Disease	Fully-adjusted model	cg20580578	7	HIP1	0.018	4.29E-03	2.10E-05
Robert F Hillary	37410739	Incident Chronic Kidney Disease	Basic model	cg20580578	7	HIP1	0.017	4.03E-03	3.20E-05
Robert F Hillary	37410739	Incident Chronic Kidney Disease	Basic model	cg20596640	13	ZMYM5	-0.013	2.92E-03	1.40E-05
Robert F Hillary	37410739	Prevalent Chronic Kidney Disease (Estimated)	Basic model	cg22959742	10	FRMD4A	0.074	1.24E-02	2.00E-09
Robert F Hillary	37410739	Prevalent Chronic Kidney Disease (Estimated)	Fully-adjusted Model	cg22959742	10	FRMD4A	0.06	1.28E-02	3.20E-06
Robert F Hillary	37410739	Prevalent Chronic Kidney Disease (Estimated)	Basic model	cg23309998	1	CHIT1	0.064	1.48E-02	1.50E-05
Robert F Hillary	37410739	Prevalent Chronic Kidney Disease (Estimated)	Fully-adjusted Model	cg23316449	14	PAPLN	0.04	9.59E-03	3.50E-05
Robert F Hillary	37410739	Incident Chronic Kidney Disease	Basic model	cg23570810	11	IFITM1	-0.013	2.73E-03	8.00E-07
Robert F Hillary	37410739	Prevalent Chronic Kidney Disease (Estimated)	Basic model	cg23570810	11	IFITM1	-0.045	8.43E-03	1.00E-07
Robert F Hillary	37410739	Prevalent Chronic Kidney Disease (Estimated)	Basic model	cg24199203	19	NFIC	0.053	1.10E-02	1.20E-06
Robert F Hillary	37410739	Prevalent Chronic Kidney Disease (Estimated)	Fully-adjusted Model	cg24199203	19	NFIC	0.05	1.14E-02	1.00E-05
Robert F Hillary	37410739	Prevalent Chronic Kidney Disease (Estimated)	Basic model	cg25569341	2	HDAC4	-0.022	5.58E-03	6.10E-05
Robert F Hillary	37410739	Prevalent Chronic Kidney Disease (Estimated)	Basic model	cg25675800	19	RINL	0.039	9.11E-03	1.70E-05
Robert F Hillary	37410739	Prevalent Chronic Kidney Disease (Estimated)	Basic model	cg27263465	2	HDAC4	0.049	1.19E-02	3.60E-05
Robert F Hillary	37410739	Prevalent Chronic Kidney Disease (Estimated)	Basic model	cg27370104	6	TRERF1	0.088	1.99E-02	1.10E-05

Supplementary Table 13A: Presence of cis methylation quantitative trait loci (mQTL) that annotated to CKD-associated CpGs

	Discovery		mQTLs presence	
Cgs	Chrom Pos	Nearest gene	GENOA	GoDMC
cg14849578	12	*SCARB1*	yes	yes
cg04725636	8	*DNAJC5B*	yes	no
cg06368300	17	*C1QL1*	yes	Yes
cg18633191	4	*C4orf50*	yes	No
cg13958199	9	*NEK6*	yes	No
cg05245822	18	*LINC01901*	yes	No
cg22959742	10	*FRMD4A*	yes	Yes
cg15716304	3	*CAPS2*	yes	No
cg09263059	17	*NMT1*	no	Yes
cg24791666	3	*CSRNP1*	yes	Yes
cg06720945	11	*PLEKHA7*	yes	No
cg12769599	11	*RAB38*	yes	Yes
cg05348871	11	*PRR5L*	yes	Yes
cg01936957	12	*NCOR2*	yes	no

Supplementary Table 13B: CKD-associated CpGs published by Schlosser et al that were replicated and presence of mQTLs in GENOA/GoDMC

CKD	Schlosser et al		meQTLs presence	
Cgs	Chrom Pos	Nearest Gene	GENOA	GODMC
cg00501876	3	*CSRNP1*	Yes	Yes
cg02304370	11	*PHRF1*	No	Yes
cg04578362	2	*TEX41*	Yes	Yes
cg06158227	15	*ZSCAN29*	Yes	Yes
cg14029001	6	*CCND3*	Yes	Yes
cg17944885	19	*ZNF788*	No	Yes

Supplementary Table 14: Traits associated with the seven unique genes that annotated to methylation quantitative trait loci from the GWAS 2023 catalog

Term	Overlap	P-value	Adjusted P-value	Odds Ratio	Combined Score	Genes
Craniofacial Microsomia	1/8	0.002	0.028	588.171	3565.886	*DCAKD*
Recurrent Major Depressive Disorder	1/12	0.003	0.028	374.218	2117.208	*TRIM55*
Systolic Blood Pressure (Weighted GWA)	1/17	0.005	0.028	257.213	1365.793	*DCAKD*
White Matter Hyperintensities	1/20	0.006	0.028	216.568	1114.856	*DCAKD*
White Matter Hyperintensity Volume X Hypertension Interaction (2Df)	1/25	0.007	0.028	171.408	844.235	*DCAKD*
Cognitive Decline Rate In Late Mild Cognitive Impairment	1/34	0.010	0.028	124.606	575.542	*TTC21A*
White Matter Hyperintensity Volume (Adjusted For Hypertension)	1/35	0.010	0.028	120.935	555.096	*DCAKD*
White Matter Hyperintensity Volume	1/44	0.013	0.030	95.581	416.952	*DCAKD*
White Matter Microstructure (Mean Diusivities)	1/51	0.015	0.031	82.172	346.395	*DCAKD*
Systolic Blood Pressure (Standard GWA)	1/76	0.022	0.042	54.715	208.989	*DCAKD*
Gut Microbiota (Bacterial Taxa, Hurdle Binary Method)	1/144	0.041	0.071	28.601	91.203	*DCAKD*
Metabolic Biomarkers (Multivariate Analysis)	1/160	0.046	0.072	25.703	79.303	*DCAKD*

Supplementary References:

1. Xu Z, Niu L, Li L, Taylor JA. ENmix: a novel background correction method for Illumina HumanMethylation450 BeadChip. Nucleic Acids Res. 2016;44(3):e20.
2. Aryee MJ, Jaffe AE, Corrada-Bravo H, Ladd-Acosta C, Feinberg AP, Hansen KD, et al. Minfi: a flexible and comprehensive Bioconductor package for the analysis of Infinium DNA methylation microarrays. Bioinformatics. 2014;30(10):1363-9.
3. Chen YA, Lemire M, Choufani S, Butcher DT, Grafodatskaya D, Zanke BW, et al. Discovery of cross-reactive probes and polymorphic CpGs in the Illumina Infinium HumanMethylation450 microarray. Epigenetics. 2013;8(2):203-9.
4. Pidsley R, Zotenko E, Peters TJ, Lawrence MG, Risbridger GP, Molloy P, et al. Critical evaluation of the Illumina MethylationEPIC BeadChip microarray for whole-genome DNA methylation profiling. Genome Biol. 2016;17(1):208.
5. Miles B. Illumina methylation array probe filtering (450k and EPIC/850k [Available from: https://github.com/sirselim/illumina450k_filtering. Accessed 15 June 2022.
6. Chen J, Hui Q, Wang Z, Wilson FP, So-Armah K, Freiberg MS, et al. Epigenome-Wide Meta-Analysis Reveals Differential DNA Methylation Associated With Estimated Glomerular Filtration Rate Among African American Men With HIV. Kidney Int Rep. 2023;8(5):1076-86.
7. Houseman EA, Accomando WP, Koestler DC, Christensen BC, Marsit CJ, Nelson HH, et al. DNA methylation arrays as surrogate measures of cell mixture distribution. BMC Bioinformatics. 2012;13:86.
8. Horvath S. DNA methylation age of human tissues and cell types. Genome Biol. 2013;14(10):R115.
9. van Iterson M, van Zwet EW, Consortium B, Heijmans BT. Controlling bias and inflation in epigenome- and transcriptome-wide association studies using the empirical null distribution. Genome Biol. 2017;18(1):19.
10. Lu AT, Quach A, Wilson JG, Reiner AP, Aviv A, Raj K, et al. DNA methylation GrimAge strongly predicts lifespan and healthspan. Aging (Albany NY). 2019;11(2):303-27.
11. Schlosser P, Tin A, Matias-Garcia PR, Thio CHL, Joehanes R, Liu H, et al. Meta-analyses identify DNA methylation associated with kidney function and damage. Nat Commun. 2021;12(1):7174.
12. Breeze CE, Batorsky A, Lee MK, Szeto MD, Xu X, McCartney DL, et al. Epigenome-wide association study of kidney function identifies trans-ethnic and ethnic-specific loci. Genome Med. 2021;13(1):74.
13. Xie Z, Bailey A, Kuleshov MV, Clarke DJB, Evangelista JE, Jenkins SL, et al. Gene Set Knowledge Discovery with Enrichr. Curr Protoc. 2021;1(3):e90.
14. Sollis E, Mosaku A, Abid A, Buniello A, Cerezo M, Gil L, et al. The NHGRI-EBI GWAS Catalog: knowledgebase and deposition resource. Nucleic Acids Res. 2023;51(D1):D977-D85.
15. Sudlow C, Gallacher J, Allen N, Beral V, Burton P, Danesh J, et al. UK biobank: an open access resource for identifying the causes of a wide range of complex diseases of middle and old age. PLoS Med. 2015;12(3):e1001779.
16. Battram T, Yousefi P, Crawford G, Prince C, Sheikhali Babaei M, Sharp G, et al. The EWAS Catalog: a database of epigenome-wide association studies. Wellcome Open Res. 2022;7:41.

17. Shang L, Zhao W, Wang YZ, Li Z, Choi JJ, Kho M, et al. meQTL mapping in the GENOA study reveals genetic determinants of DNA methylation in African Americans. Nat Commun. 2023;14(1):2711.
